# Robotic versus laparoscopic TME for rectal cancer: meta-analysis of pathological quality indicators

**DOI:** 10.1007/s10151-026-03369-7

**Published:** 2026-05-29

**Authors:** S. Morarasu, S. Lunca, C. Clancy, W. L. Ong, E. Morpurgo, G.-M. Dimofte

**Affiliations:** 1 Grigore T Popa University of Medicine and Pharmacy Iasi, Iasi, Romania; 2 2nd Department of Surgical Oncology, Regional Institute of Oncology (IRO), Iasi, Romania; 3 Department of Colorectal Surgery, Tallaght University Hospital, Dublin 24, Ireland; 4 Trinity College Dublin, Dublin, Ireland; 5 Regional Center for Videolaparoscopic Robotic Surgery, Ospedale di Camposampiero, Azienda ULSS 6 Euganea, Camposampiero, Italy

**Keywords:** Rectal cancer, Robotic surgery, Laparoscopic surgery, Total mesorectal excision, Anterior resection

## Abstract

**Background:**

Total mesorectal excision (TME) quality is a key determinant of oncological outcomes in rectal cancer. While robotic surgery offers technical advantages over laparoscopy in the confined pelvis, its superiority regarding pathological outcomes remains debated. We conducted a meta-analysis comparing robotic and laparoscopic TME focusing on quality indicators of TME and risk factors for incomplete TME.

**Methods:**

A PROSPERO-registered systematic search of PubMed and EMBASE up to May 2025. Comparative studies reporting pathological outcomes of robotic versus laparoscopic TME were included. Primary endpoints were TME completeness, circumferential resection margin (CRM) positivity and distal resection margin (DRM) positivity. Secondary analysis included baseline characteristics (male gender, BMI, bulky tumours, distance to anal verge, neoadjuvant radiotherapy) and local recurrence rate.

**Results:**

Fifty-six studies (27,648 patients; robotic 10,629, laparoscopic 17,019) were included. Robotic surgery was associated with significantly more complete TME specimens (OR 1.50, 95% CI 1.23–1.82, *p* < 0.001) and fewer positive DRMs (OR 0.68, 95% CI 0.48–0.97, *p* = 0.031). CRM positivity was comparable between groups (OR 0.93, 95% CI 0.77–1.12, *p* = 0.44). In random-effects analysis, there was a non-significant trend towards fewer local recurrences after robotic TME (OR 0.75, 95% CI 0.54–1.05, *p* = 0.09). Robotic cohorts more frequently included male patients, distal tumours and neoadjuvant chemoradiotherapy, suggesting preferential selection of technically challenging cases.

**Conclusions:**

Robotic TME is associated with higher specimen completeness and lower DRM positivity compared with laparoscopic TME, while CRM positivity and local recurrence rates appear broadly similar. These data support the use of robotics as a primary minimally invasive option for mid–low rectal cancer; however, as a result of low certainty of evidence, these findings should be interpreted cautiously.

**Synopsis:**

This meta-analysis provides the most up-to-date synthesis of pathological outcomes comparing robotic and laparoscopic TME for rectal cancer, incorporating data from 56 studies including the recent REAL and COLRAR randomized trials. Unlike previous reviews, it exclusively analyses *total* mesorectal excision procedures, excluding partial or high anterior resections, thereby eliminating a major source of heterogeneity. The findings demonstrate that robotic TME is associated with higher specimen completeness and fewer positive distal margins, even in technically demanding mid- and low-rectal cancers, supporting the role of robotics as the preferred minimally invasive approach in mid–low, difficult tumours.

**Supplementary Information:**

The online version contains supplementary material available at 10.1007/s10151-026-03369-7.

## Introduction

Total mesorectal excision (TME) is the pinnacle of rectal cancer treatment offering, alongside chemoradiotherapy, excellent local recurrence rates for mid–low rectal tumours. Since its introduction by Heald et al., TME has become the reference point for quality in rectal cancer surgery [1, 2]. Ensuring a complete TME specimen by respecting the mesorectal fascia and achieving negative circumferential (CRM) and distal resections margins (DRM) in more than 90% of cases are now widely recognized as quality markers of excellence for rectal cancer centres [3, 4]. Traditionally performed through an open approach, TME has evolved with the advent of minimally invasive surgical techniques, most notably laparoscopic and, more recently, robotic surgery.

Laparoscopic TME has demonstrated several short-term benefits over open surgery, including reduced blood loss, faster postoperative recovery, and shorter hospital stay [5–11]. However, the steep learning curve, limited instrument articulation, unstable view and technical challenges in the confined pelvic space are important limitations. While in expert hands laparoscopic surgery can match open surgery in terms of specimen quality, some randomized controlled trials (RCTs) failed to prove the non-inferiority of laparoscopic surgery [9–12]. To overcome limitations of laparoscopic approach, robotic surgery has emerged as a solid alternative, offering the best of both worlds: faster recovery, less morbidity but also stable, clear view and comfortable dissection in the narrow pelvis, which should translate into superior specimens.

The technical advantages of robotic surgery are obvious and supported by a low reversion rate to laparoscopy once surgeons become comfortable using the robotic platform. Despite this, robotic surgery is yet to have consistent institutional endorsement for implementation in many cancer centres. Alongside the increased costs another argument against the wide adoption of robotic surgery is the lack of solid data to prove the superiority of robotic surgery when it comes to pathological and oncological outcomes. The pivotal ROLARR trial [13] is usually given as an example, as it showed similar TME completeness and CRM positivity rate between robotic and laparoscopic TME; however, a secondary sensitivity analysis (ROLARR 2) [14] found that the results may have been affected by a learning curve effect of robotic surgery and once surgeons surpass the learning curve (i.e. more than 40 cases) pathological outcomes (TME completeness, CRM+ rate) may be in favour of robotic surgery, although not always statistically significant.

Clearly there is still debate regarding the superiority of robotic surgery in achieving better pathological outcomes when used for TME surgery. For this reason and to better stratify the role of the robotic platform in the management of rectal cancer we have conducted a large meta-analysis comparing pathological outcomes between robotic versus laparoscopic TME.

## Materials and methods

### Literature search and study selection

**Fig. 1:**
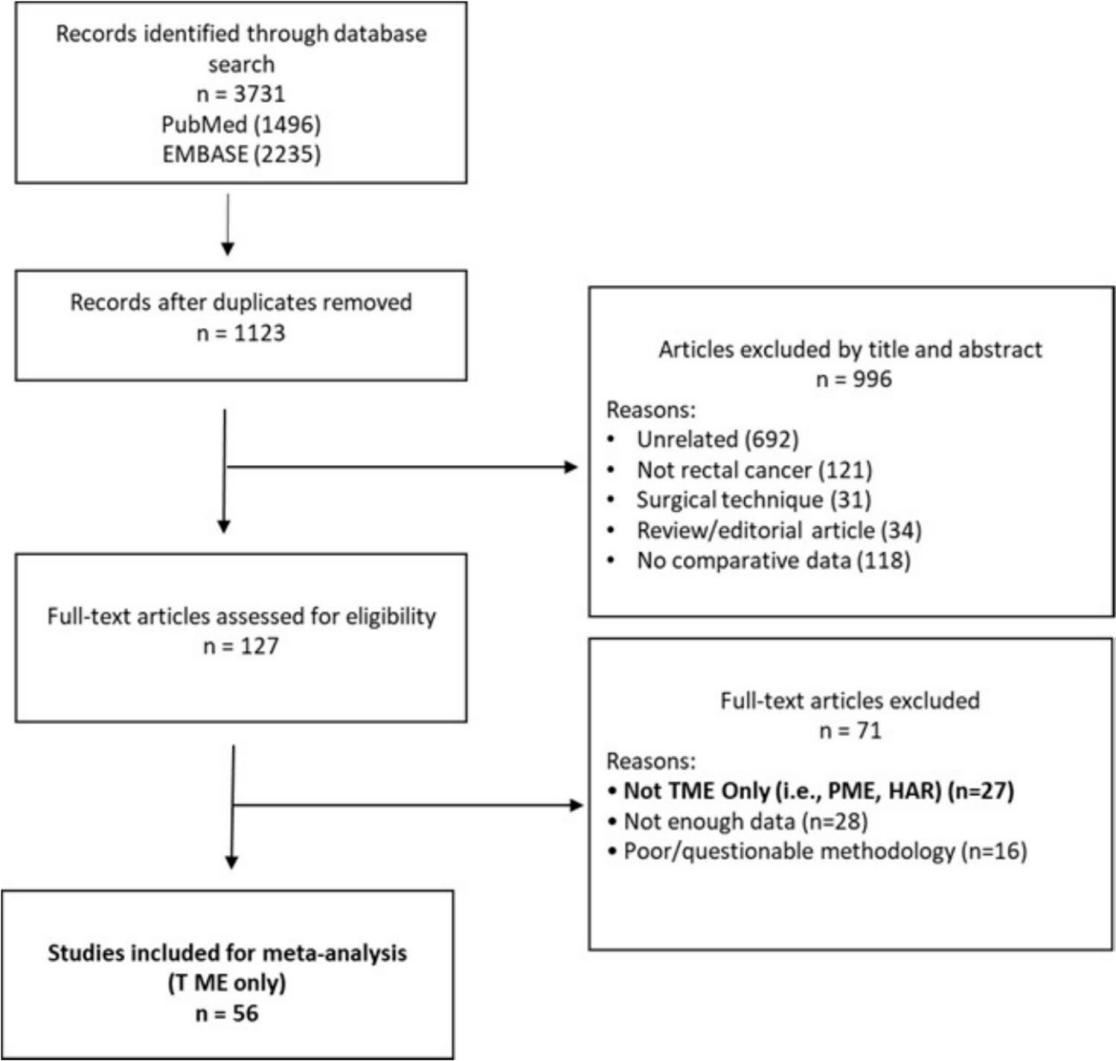
PRISMA flowchart

The study was registered with PROSPERO (International Prospective Register of Systematic Reviews) [15]. The study ID is CRD420251024701. A systematic search of PubMed and EMBASE databases was performed for all comparative studies examining pathological outcomes in patients that underwent robotic versus laparoscopic TME for rectal cancer. In addition, reference lists of eligible articles and relevant reviews were hand-searched to identify further studies. The following search algorithm was used: (robotic) AND (laparoscopic) AND (rectal cancer OR total mesorectal excision OR anterior resection). Preferred Reporting Items for Systematic Reviews and Meta-Analyses (PRISMA) guidelines [16] were used as search protocol and the PRISMA checklist was followed to conduct the methodology (Fig. 1). Inclusion criteria were used according to the Problem, Intervention, Comparison, Outcome and Study Design (PICOS) formula. The population consisted of patients undergoing TME for rectal cancer either by robotic (I) or laparoscopic approach (C). The primary endpoint was TME quality (completeness of mesorectal excision). Secondary endpoints included CRM positivity, distal resection margin (DRM) positivity, baseline tumour characteristics (gender, BMI, tumour stage, distance from anal verge, neoadjuvant therapy), and local recurrence. Regarding study design, RCTs and observational comparative studies were included. The latest search was performed on 30 May 2025. Two authors (SM and WLO) assessed the titles and abstracts of studies found in the search and the full texts of potentially eligible trials were reviewed. Disagreements were resolved by consensus-based discussion. The ROB2 [17], ROBINS-I tools [18] and GRADE assessment (Fig. S1 and Table 2) were used to quantify quality of eligible studies as previously done [19, 20]. The references of full texts reviewed were further screened for additional eligible studies. The corresponding author was contacted to clarify data extraction if additional information was necessary. Attempt was made to contact authors of studies excluded because TME was not the single intervention (i.e. partial mesorectal excision, high anterior resection). Response rate was unsatisfactory.

### Eligibility criteria

Studies written in English including comparative pathological data between robotic versus laparoscopic were assessed for eligibility. After initial triage, studies with poor methodology and high risk of bias were excluded from the final analysis. The primary endpoints were TME quality (mesorectal/intramesorectal/muscularis propria or reporting as complete/non-complete), CRM+ rate and DRM+ rate. Secondary analysis included comparison of baseline tumour characteristics (i.e. staging, distance from anal verge, neoadjuvant therapy) and local recurrences (LR). Studies that did not provide data on TME quality or CRM+ rate were excluded. Studies without comparative data were not included. Studies that compared robotic TME vs open TME were excluded. Studies that included partial mesorectal excisions (PME) or high anterior resections (HAR) were excluded. Retrospective studies that did not report on the tumour characteristics between the two groups were excluded because of the unacceptable risk of selection bias.

### Data extraction and outcomes

For each eligible study the following data was recorded: author’s names, journal, year of publication, study type, total number of patients and number of patients included in each group, mean age, mean BMI, tumour staging, distance from anal verge, neoadjuvant therapy, type of surgery, previous abdominal surgery, TME quality, CRM+ rate, DRM+ rate, lymph node yield, R0/R1/R2 resection rate, local recurrences. For each study, the outcomes of interest were extracted and grouped into two main categories which were further analysed: (i) baseline characteristics (BMI, male gender, tumour staging, neoadjuvant therapy, distance from anal verge); (ii) pathological outcomes (TME completeness, CRM+ rate, DRM+ rate, local recurrence rate).

### Statistical analysis

For dichotomous outcomes, pooled odds ratios (ORs) with 95% confidence intervals (CIs) were calculated. Continuous outcomes were synthesised as mean differences with 95% CIs, using standard methods for imputation of missing standard deviations where necessary. Random-effects models as described by DerSimonian–Laird [21] were specified a priori as the primary analytic approach for all outcomes, given the expected clinical and methodological heterogeneity across studies. Fixed-effects models were used only in prespecified sensitivity analyses when between-study heterogeneity was low and are reported descriptively.

Statistical heterogeneity was assessed using Cochran’s *Q* test [22] and quantified with the *I*^2^ statistic. We considered *I*^2^ values of 25%, 50% and 75% to correspond to low, moderate and high heterogeneity, respectively.

For each outcome, forest plots display individual study estimates (squares proportional to study weight) and pooled estimates (diamonds). Where at least ten studies contributed data to a given outcome, we visually inspected funnel plots of log(OR) against standard error to explore the possibility of small-study effects and publication bias. We did not perform formal regression-based tests for funnel plot asymmetry for binary outcomes, given their known limitations in this setting. To explore whether tumour height influenced distal margin outcomes, we performed a random-effects meta-regression using study-level mean distance from the anal verge as a moderator. All analyses were performed using RevMan v5.3 [23] and MedCalc v23.2.0 [24].

## Results

### Eligible studies

Fifty-six studies [13, 25–79] containing data comparing robotic vs laparoscopic TME were included (Table 1). The initial search found 3731 studies. After excluding duplicates and unrelated studies based on abstract triage, 127 full texts were assessed for eligibility, out of which 56 matched the inclusion criteria and were systematically reviewed (Fig. 1). Twenty-seven studies were excluded because TME was not the only procedure in the two groups (i.e. partial mesorectal excision or high anterior resection). The year of publication of included studies ranged from 2013 to 2025. The total number of included patients was 27,648, split into two groups: study group (ROB, *n* = 10,629) and control group (LAP, *n* = 17,019). Four RCTs and 19 case-matched studies were included. All robotic procedures were carried out using the da Vinci Si or Xi system. Standard TME technique was adhered to in both groups. The mean age in the ROB group was 61.9, SD = 10.2 vs 62.3, SD = 10.3 in the LAP group. Mean BMI was 24.5, SD = 3.1 in the ROB group and 24.4, SD = 3.2 in the LAP group.

**Table 1:** Overview of included studies

Author	Year	Type	Bias	No. of patients	ROB no.	LAP no.	ROB age (years) Mean	SD	LAP age (years) Mean	SD	ROB male patients	LAP male patients
Aliyev	2023	Retrospective	Serious	68	34	34	58.0	6.2	57.0	8.6	NR	NR
Angehrn	2022	Prospective	Moderate	102	38	64	66.0	5.5	63.0	4.0	29	42
Aselmann	2018	Retrospective	Serious	85	44	41	61.1	11.5	65.1	12.0	26	24
Asoglu	2019	Retrospective	Serious	79	14	65	54.0	7.5	57.0	13.0	14	65
Baek	2011	CM	Moderate	82	41	41	63.6	9.7	63.7	11.5	25	25
Barnajian	2013	CM	Serious	40	20	20	62.0	9.5	63.0	11.2	12	12
Burghgraef	2022	CM	Moderate	630	315	315	67.1	10.6	67.4	9.4	194	196
Chen	2020	Retrospective	Serious	125	88	37	62.0	12.1	60.0	11.1	54	20
Chen	2022	CM	Moderate	112	56	56	57.4	11.2	56.4	10.9	39	38
Cho	2015	CM	Moderate	556	278	278	57.4	11.6	58.3	10.4	182	184
Feng (REAL)	2025	RCT	Low	1171	586	585	59.1	11.0	60.7	9.8	356	354
Feroci	2016	Retrospective	Serious	111	53	58	66.0	10.5	66.0	11.7	27	42
Guo	2024	CM	Moderate	662	331	331	NR	NR	NR	NR	207	203
Horsey	2022	CM	Moderate	3782	1891	1891	73.1	6.4	74.0	6.8	1204	1176
Hu	2020	Retrospective	Serious	3845	1164	2681	56.5	15.0	53.4	16.0	688	1427
Huang	2017	Retrospective	Serious	78	40	38	60.0	12.2	60.1	14.2	25	28
Ielpo	2017	Retrospective	Serious	198	86	112	63.9	9.5	61.6	11.9	48	67
Jayne (ROLARR)	2018	RCT	Low	471	237	234	64.4	10.9	65.5	11.9	161	159
Kamali	2017	Retrospective	Serious	36	18	18	73.0	11.0	66.0	11.0	13	11
Kim	2016	CM	Serious	99	33	66	57.0	9.6	58.2	9.8	23	46
Kim	2017	CM	Moderate	448	224	224	60.7	11.7	61.0	11.0	145	141
Kim	2017	RCT	Low	139	66	73	60.4	9.7	59.7	11.7	51	52
Law	2016	Retrospective	Serious	391	220	171	65.0	14.0	67.0	18.2	148	97
Lei	2021	Retrospective	Serious	534	314	220	58.9	12.4	58.8	12.4	194	146
Li	2022	Retrospective	Serious	275	148	127	61.4	8.8	59.6	10.5	96	78
Li	2023	CM	Moderate	420	210	210	62.3	10.5	62.5	10.8	119	121
Lim	2016	Retrospective	Serious	138	74	64	65.1	12.4	65.8	11.1	50	46
Lim	2023	Retrospective	Serious	110	46	64	61.0	11.4	63.0	10.4	29	43
Liu	2022	Retrospective	Serious	92	47	45	58.7	7.6	58.5	8.7	27	25
Mirza	2024	Retrospective	Serious	313	159	154	60.0	4.0	60.0	2.0	99	92
Olthof	2020	Retrospective	Serious	320	100	220	68.0	4.0	69.0	3.7	63	141
Pan	2022	Retrospective	Serious	106	56	50	64.0	3.2	64.0	2.7	53	43
Park	2015	Retrospective	Serious	217	133	84	59.2	11.4	63.5	11.2	86	60
Park	2021	CM	Moderate	236	118	118	60.0	10.8	60.3	11.1	90	87
Park (COLRAR)	2023	RCT	Low	295	151	144	65.5	11.4	67.2	10.1	97	99
Ramji	2015	Retrospective	Serious	53	26	27	62.1	9.1	63.7	11.2	19	19
Rogier-Mouzelas	2024	CM	Moderate	120	60	60	67.0	4.0	68.0	4.0	45	44
Rouanet	2018	Retrospective	Moderate	400	200	200	64.0	15.0	63.5	12.7	131	136
Rutgers	2024	Retrospective	Serious	490	173	317	68.0	10.6	67.0	9.9	109	197
Saklani	2013	Retrospective	Serious	138	74	64	59.6	12.3	60.1	10.8	50	46
Shadmanov	2024	Retrospective	Serious	672	183	489	54.2	12.8	57.2	12.2	138	280
Silva-Velazco	2016	Retrospective	Serious	184	66	118	59.0	12.0	60.0	14.7	50	66
Song	2021	Retrospective	Serious	99	70	29	57.5	12.5	60.0	11.5	46	24
Speicher	2015	Retrospective	Serious	6403	956	5447	59.0	4.5	61.0	4.7	562	3167
Sueda	2021	CM	Moderate	200	100	100	70.5	10.9	70.1	10.2	59	60
Sugoor	2019	CM	Moderate	168	84	84	48.3	15.5	49.2	14.8	61	58
Sun	2024	CM	Moderate	136	68	68	62.5	8.7	63.1	8.0	48	46
Tejedor	2019	CM	Moderate	272	136	136	68.0	16.0	69.0	14.0	76	76
TengTeng	2025	Retrospective	Serious	102	51	51	53.0	6.0	54.0	4.0	30	32
Tilney	2021	Retrospective	Serious	337	204	133	64.6	11.4	66.6	12.2	157	83
Valverde	2017	Retrospective	Serious	130	65	65	67.0	11.0	65.0	10.0	42	45
Wang	2025	CM	Moderate	564	282	282	NR	NR	NR	NR	177	181
Yamaguchi	2015	Retrospective	Serious	442	203	239	64.8	10.8	65.9	10.8	140	154
Yamanashi	2022	CM	Moderate	60	30	30	62.4	11.0	61.7	10.3	24	26
Yoo	2015	Retrospective	Serious	70	44	26	59.7	12.3	60.5	10.7	35	19
Zhao	2024	CM	Moderate	242	121	121	NR	NR	NR	NR	67	66

Bias assessment done according to ROBINS-I/ROB2 tools depending on the type of study observational/RCT. Detailed risk of bias assessment depicted in Suppl. Fig. S1

*CM* case matched, *RCT* randomized controlled trial, *ROB* robotic, *LAP* laparoscopic, *SD* standard deviation, *NR* not recorded

### Baseline characteristics

#### Gender

Fifty-five studies describing 27,580 patients included data on patients’ gender. Overall, there were fewer male patients included in the LAP group, (OR 1.085, 95% CI 1.029–1.144, *p* = 0.003) with no significant inter-study heterogeneity (Chi^2^ = 64.44, *df* = 53, *p* = 0.134, *I*^2^ = 17.7%) (Fig. S2). Funnel plot (Fig. S3) showed no significant asymmetry suggestive of publication bias (*p* = 0.436, 95% CI 0.318–0.726).

#### BMI

Forty-six studies including 14,250 patients compared the mean BMI between the two groups. Mean BMI was similar among the two groups with a mean difference of 0.0519, 95% CI 0.008–0.112, *p* = 0.090 with significant inter-study heterogeneity (Chi^2^ = 108.1, *df* = 45, *p* < 0.0001, *I*^2^ = 58.39%) (Fig. S4). Funnel plot (Fig. S5) showed no significant publication bias (*p* = 0.223, 95% CI 0.369–1.538).

#### T3/4 tumours

Thirty-two studies including data from 12,918 patients had pathological data regarding the rate of pT3/4 tumours in each group. There were no differences in the number of pT3/4 tumours between the two groups (OR 0.906, 95% CI 0.783–1.049, *p* = 0.186). Significant heterogeneity was found among studies (Chi^2^ = 79.98, *df* = 31, *I*^2^ = 61.24%) (Fig. S6). No significant publication bias was found (*p* = 0.532, 95% CI 1.012–1.918) (Fig. S7).

#### N+ tumours

Thirty studies with 8988 patients provided data on N status (positive/negative) in the two groups. The two groups were similar in this regard (OR 0.997, 95% CI 0.833–1.193, *p* = 0.971) with significant inter-study heterogeneity (Chi^2^ = 96.54, *df* = 29, *I*^2^ = 69.96%) (Fig. S8). No significant publication bias was found (*p* = 0.086, 95% CI 0.212–3.009) (Fig. S9).

#### Neoadjuvant therapy

Fifty-two studies including data on 26,643 patients (10,069, ROB vs 16,574, LAP) stated the number of patients that received neoadjuvant chemoradiotherapy in each group (regardless of protocol used, i.e. short-course/long-course/total neoadjuvant therapy). Significantly more patients that received neoadjuvant therapy were included in the ROB group (OR 1.382, 95% CI 1.181–1.617, *p* < 0.001). Significant inter-study heterogeneity was found (Chi^2^ = 208.91, *df* = 42, *I*^2^ = 79.90%) (Fig. S10). No significant publication bias was found (*p* = 0.872, 95% CI 1.024–1.203) (Fig. S11).

#### Distance from anal verge

Thirty-three studies including data on 7701 patients (3854, ROB vs 3847, LAP) compared the two groups in terms of distance from the anal verge to the lower pole of the tumour. Tumours included in the robotic group had significantly lower distance to the AV compared to LAP (MD 0.125 cm, 95% CI 0.007–0.242, *p* < 0.037) with significant inter-study heterogeneity present (Chi^2^ = 187.90, *df* = 32, *I*^2^ = 82.97%) (Fig. S12). Funnel plot did not show significant asymmetry suggestive of publication bias (*p* = 0.904, 95% CI 1.991–2.241) (Fig. S13).

### Pathological outcomes

#### Circumferential resection margin

Fifty-six studies including data on 27,592 patients (10,604, ROB vs 16,988, LAP) compared circumferential resection margin positivity (CRM+) between the two groups. There was no significant difference in CRM+ rates (OR 0.930, 95% CI 0.774–1.118, *p* = 0.441) with low inter-study heterogeneity (Chi^2^ = 68.61, *df* = 51, *I*^2^ = 25.67%) (Fig. S14). Funnel plot did not show significant publication bias (*p* = 0.988, 95% CI − 0.453 to 0.446) (Fig. S15).

#### Distal resection margin

Fourteen studies including data on 8283 patients (3381, ROB vs 4902, LAP) provided data on the rate of distal resection margin positivity (DRM+) in the two groups. Significantly fewer patients had positive DRM in the ROB group (OR 0.678, 95% CI 0.477–0.965, *p* = 0.031) with no inter-study heterogeneity (Chi^2^ = 8.55, *df* = 13, *I*^2^ = 0%) (Fig. S16). Funnel plot did not show significant publication bias (*p* = 0.355, 95% CI 0.499–1.289) (Fig. S17). To reduce case-mix confounding, we performed meta-regression using DAV (mean difference between ROB group and LAP group) as a covariate. Meta-regression demonstrated no significant association between tumour height and the effect size for distal resection margin positivity (β = 0.24, SE 0.69; *p* = 0.73), indicating that differences in tumour height did not explain between-study variation in DRM outcomes.

#### TME quality

**Fig. 2:**
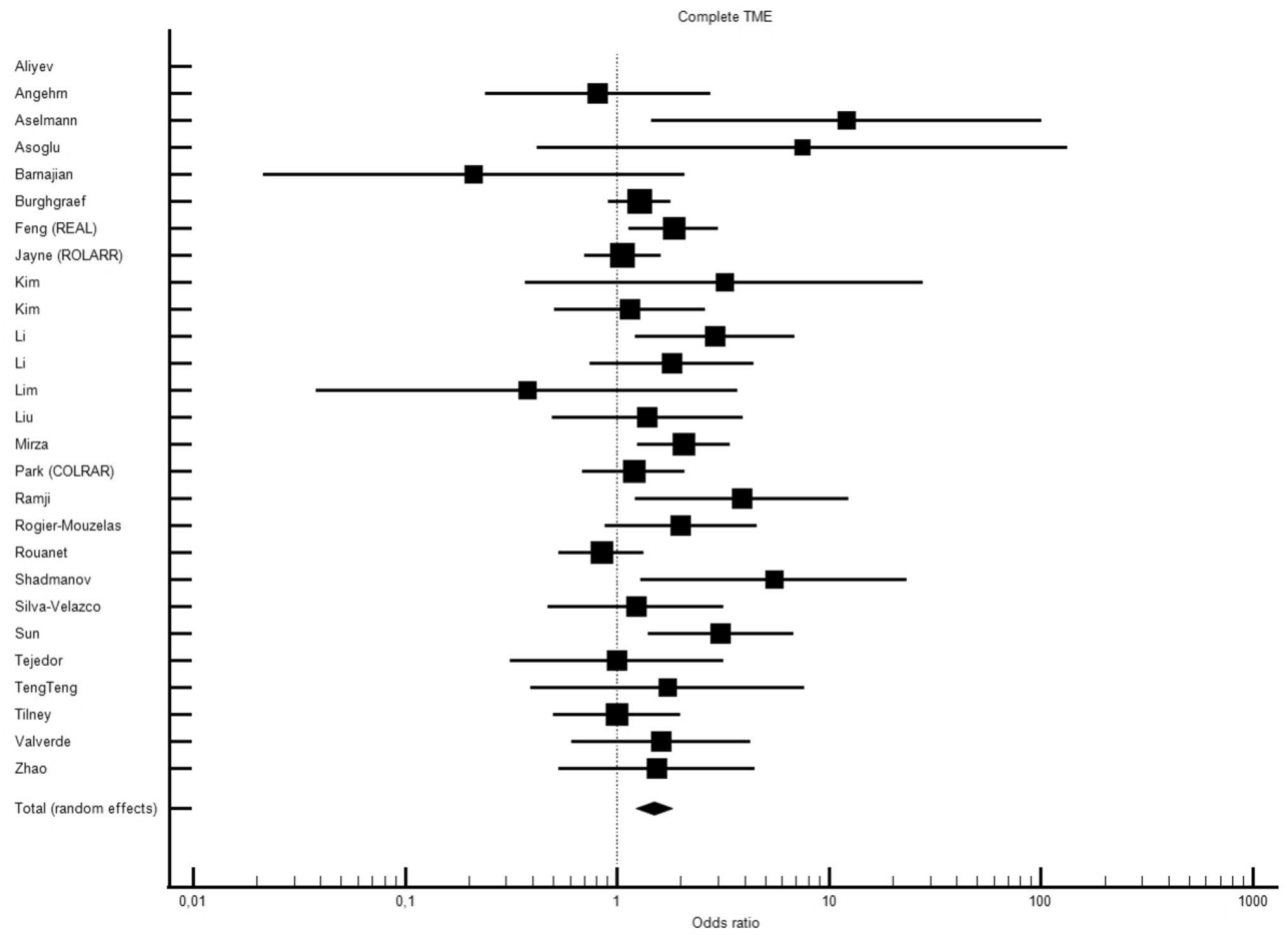
ROB vs LAP and quality of TME

Twenty-seven studies including data on 7065 patients (3356, ROB vs 3709, LAP) provided data on the quality of TME (complete/incomplete/mesorectal/intramesorectal/muscularis propria) between ROB vs LAP. Robotic surgery was associated with significantly more complete TME specimens compared to LAP (OR 1.497, 95% CI 1.229–1.822, *p* < 0.001) with moderate inter-study heterogeneity (Chi^2^ = 36.60, *df* = 25, *I*^2^ = 31.70%) (Fig. 2). Funnel plot did not show significant asymmetry suggestive of publication bias (*p* = 0.1334, 95% CI 0.242–1.717) (Fig. S18).

#### Local recurrences

**Fig. 3:**
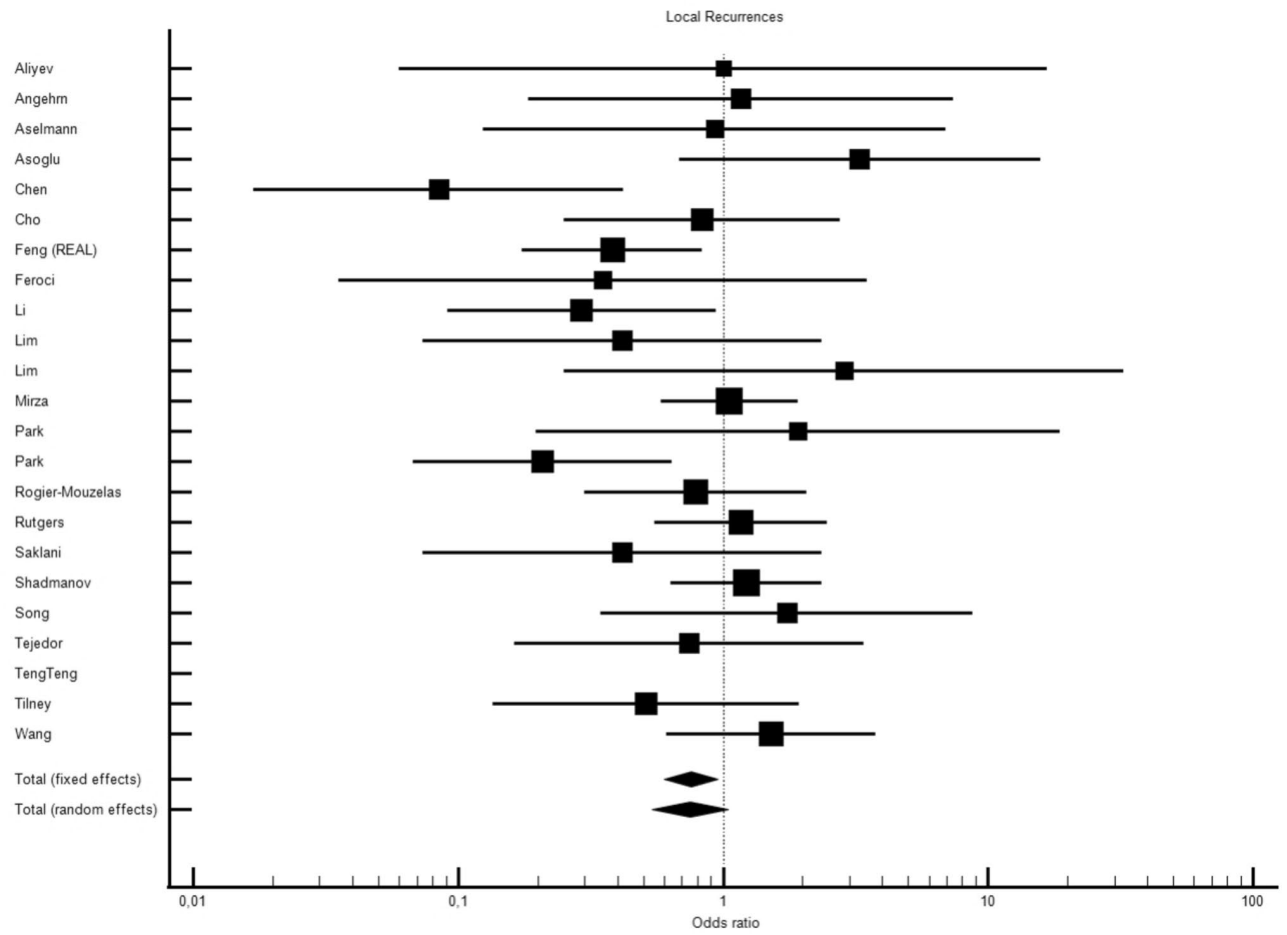
ROB vs LAP and local recurrences

Twenty-three studies including data on 6360 patients (3038, ROB vs 3322, LAP) reported the number of local recurrences (LR) in each group. Mean follow-up was 46.50 ± 10.4 months in the ROB group and 47.62 ± 14.2 months in the LAP group. Despite a trend towards fewer recurrences in the ROB group, mostly visible in a fixed-effects model (OR 0.757, 95% CI 0.600–0.955, *p* < 0.019), overall, in the standard random-effects model, there was no significant difference (OR 0.751, 95% CI 0.539–1.045, *p* < 0.090) with a low–moderate inter-study heterogeneity (OR 0.757, 95% CI 0.600–0.955, *p* < 0.019) (Fig. 3, Fig. S19).

### Sensitivity analysis: RCTs only

**Fig. 4:**
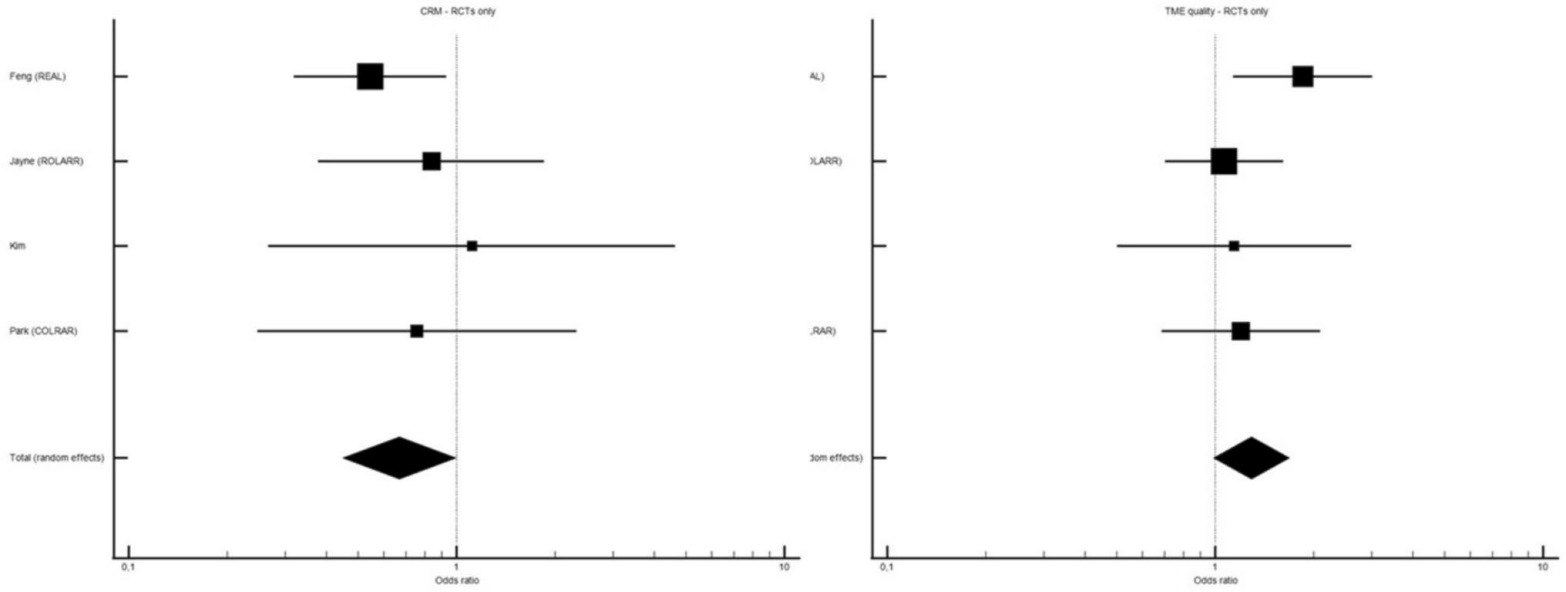
ROB vs LAP in terms of CRM positivity rate and TME completeness rate. RCTs only

Further sensitivity analysis was performed restricting to randomized trials only for available primary outcomes. A trend towards better TME quality was seen in the ROB group, without reaching statistical significance (OR 1.290, 95% CI 0.989–1.683, *p* = 0.061) while a higher rate of CRM+ was seen in the LAP group (OR 0.669, 95% CI 0.450–0.994, *p* = 0.047) (Fig. 4).

## Discussion

Our analysis shows that robotic surgery is associated with superior specimens compared to laparoscopic TME considering a higher rate of TME completeness and a lower rate of distal resection margin positivity. CRM and LR are largely similar in robotic and laparoscopic TME. However, as a result of the lack of quality in evidence as depicted by the GRADE assessment, there findings should be interpreted with caution. Noteworthy is that the above results supporting robotic surgery stand despite that male patients, lower rectal tumours and tumours with neoadjuvant radiotherapy are more frequently considered for robotic rather than laparoscopic TME which may well be a selection bias favouring laparoscopic surgery.

To our knowledge this is the first meta-analysis to compare robotic vs laparoscopic TME only (excluding partial mesorectal excision or rectosigmoid resections) in terms of baseline characteristics (which are known to increase TME difficulty) such as male gender, BMI, bulky tumours, neoadjuvant radiotherapy and lower rectum tumours (distance to AV) and in terms of pathological outcomes with focus on the most relevant ones for measuring quality in TME practice: CRM, TME completeness and DRM. Importantly, meta-regression did not identify tumour height as a significant moderator of distal margin positivity, suggesting that the observed association between robotic surgery and lower DRM positivity is not driven solely by differences in tumour location. At a glance current literature has conflicting evidence regarding the superiority of robotic surgery, both individual studies and meta-analyses published so far being heterogenous in terms of inclusion and sought endpoints. Most research showing similar outcomes between the two TME approaches focused on operative and functional outcomes such as conversion rate, operative time, morbidity, sexual/urinary dysfunction and patient-reported outcomes. Operative and functional outcomes are expected to be comparable as laparoscopic surgery is proven to be reliable in experienced hands; however, the rigid instrumentation and poor visualization in the deep pelvis makes laparoscopic surgery demanding and poses a high risk of pathological failure even if operative and functional outcomes are met. It is here where robotic surgery should have the most benefit, raising the specimen quality of minimally invasive TME in difficult cases. A recent meta-analysis by Ishizuka et al. [80] including 18 studies showed better CRM in the robotic group. Our study failed to prove this, but considering that robotic surgery was done for lower, bulkier tumours with neoadjuvant therapy and more frequent in male patients, achieving similar CRM+ rate should be regarded as a better result. A meta-analysis by Milone et al. from 2019 [81], including 12 studies, showed like our study better TME completeness for the robotic approach. The ROLARR trial [13] showed similar pathological outcomes; however, it is known to be affected by the learning curve effect as shown in a secondary sensitivity analysis. A significant proportion of surgeons might have underperformed on the robotic platform diluting any advantage on pathological outcomes. Also, the ROLARR trial included patients from 2011 to 2014 which was still during the early phase of robotic TME implementation and before the adoption of the Xi platform which made multi-quadrant surgery easier, significantly reduced external collisions and enhanced reach in the deep pelvis thanks to longer, slimmer instruments. Also, the lack of SureForm™ robotic stapler and robotic vessel sealer made rectal surgery more cumbersome in the da Vinci Si era. The more recent REAL trial [35], which was also updated in 2025 with long-term outcomes, is in favour of robotic surgery showing better CRM+ rate and better-quality specimens while in the latter secondary analysis [82] robotic surgery was found to have significantly better local recurrence rates compared to laparoscopic TME. In our study laparoscopic surgery had comparable recurrence rates to those of robotic surgery contradicting the findings of the recent REAL RCT. However, there are several factors that must be considered. The lack of statistical significance in local recurrence may relate to the use of a random-effects model. While we pre-specified random-effects models because of expected clinical heterogeneity, between-study heterogeneity for local recurrence was low. Under these conditions, a fixed-effects model—which assumes a common underlying treatment effect—may also be considered appropriate. Indeed, when applying a fixed-effects model, robotic surgery was associated with a significantly lower risk of local recurrence (OR 0.76, 95% CI 0.60–0.96; *p* = 0.019). Random-effects models are inherently more conservative, incorporating between-study variance and resulting in wider confidence intervals. Therefore, the absence of statistical significance in the primary analysis likely reflects reduced statistical power rather than true equivalence between approaches. These findings suggest a signal toward oncologic benefit with robotic surgery that may become more apparent in larger randomized trials with longer follow-up, which was the case of the REAL RCT. Secondly, we must accept that the neoadjuvant and adjuvant strategies have been refined in the last decade, especially in the form of advanced biologics and immunotherapy which could compensate for surgical technique similar to how neoadjuvant radiotherapy compensated for improper technique in the pre-TME era. We must not forget how short-course preoperative radiotherapy reduced local recurrence rate from 24% to 11% in the pre-TME era, when conventional surgery was standard [83]. Further, in the Dutch TME trial [84], TME alone, without radiotherapy, was associated with a recurrence rate of 10.9%, similar to that of conventional technique + radiotherapy. Our study shows a significant case-mix imbalance between the two approaches (more locally advanced cancers in the robotic group) which could have biased oncological results favouring laparoscopy. Despite this, robotic surgery still showed non-inferiority in terms of local control alongside superior specimens.

Our study has limitations. Our aim was to capture all real-world data on robotic vs laparoscopic TME thus including retrospective studies which clearly pose an innate risk of selection and confounding bias. As such, GRADE assessment (Table 2) showed overall low level of evidence considering that 93% of included studies were observational. More so, the data for many variables was highly heterogenous resulting in increased variance and wide confidence intervals leading to a high risk of over/underestimation of results. As an example, the definition and reporting of TME quality were not fully standardized across included studies. Although most studies used the Quirke classification to assess mesorectal plane integrity with three outcomes (complete/near complete/incomplete), others reported TME quality using binary outcomes (complete/incomplete). Pooling heterogeneous definitions may introduce misclassification bias, as specimens categorized as “complete” in one study may not be strictly equivalent to those in another. More so, although more local advanced tumours which underwent neoadjuvant therapy were allocated to the robotic group, neoadjuvant therapy itself may have had an influence on the difference in DRM+ rates. Surgical technique was reported briefly in all studies; however, variation in surgical technique and quality of dissection cannot be overlooked, especially when reporting results from multiple studies/surgeons. Similarly to the ROLARR trial, which is the only study to have considered the learning curve effect in the robotic surgery group, many of the studies included herein may have been misinterpreted in the same manner. Despite its limitations, this meta-analysis is a strong reflection of real surgical practice and the quality of robotic vs laparoscopic TME. Pathological outcomes are standardized measurements which are unlikely to be misreported considering that performance bias does not exist in retrospective studies and in the RCTs included here pathologists were blinded to the procedure. Our data shows a clear advantage for the robotic platform when discussing TME quality indicators.

**Table 2:** GRADE assessment of primary outcomes

Outcome	No. of studies	Total patients	Effect estimate (ROB vs LAP)	Absolute effect^a^	Certainty of evidence (GRADE)	Reasons for downgrading
TME completeness	27	7065	OR 1.50 (95% CI 1.23–1.82)	+ 6.1% complete TME	Low	Observational design, residual confounding
CRM positivity	56	27,592	OR 0.93 (95% CI 0.77–1.12)	− 0.4% CRM+	Low	Observational design, imprecision, potential confounding
DRM positivity	14	8283	OR 0.68 (95% CI 0.48–0.97)	− 1.2% DRM+	Low	Observational design, indirectness, potential confounding
Local recurrence	23	6360	OR 0.75 (95% CI 0.54–1.05)	− 1.5% LR	Very low	Observational design, non-standardized surveillance, limited follow-up

*TME* total mesorectal excision, *CRM* circumferential resection margin, *DRM*, distal resection margin, *ROB* robotic, *LAP* laparoscopic

## Conclusion

Robotic surgery produces superior-quality TME specimens compared to laparoscopic surgery and better distal resection margin positivity rate despite having more male patients and more distal tumours that underwent neoadjuvant therapy. Despite the overall low-quality evidence, robotics seems to be a preferred option for tackling mid–low rectal tumours.

## Supplementary Information

Below is the link to the electronic supplementary material.

Supplementary file1 (JPG 125 KB)

Supplementary file2 (JPG 191 KB)

Supplementary file3 (JPG 89 KB)

Supplementary file4 (JPG 182 KB)

Supplementary file5 (JPG 103 KB)

Supplementary file6 (JPG 154 KB)

Supplementary file7 (JPG 94 KB)

Supplementary file8 (JPG 143 KB)

Supplementary file9 (JPG 94 KB)

Supplementary file10 (JPG 179 KB)

Supplementary file11 (JPG 104 KB)

Supplementary file12 (JPG 154 KB)

Supplementary file13 (JPG 94 KB)

Supplementary file14 (JPG 210 KB)

Supplementary file15 (JPG 107 KB)

Supplementary file16 (JPG 114 KB)

Supplementary file17 (JPG 90 KB)

Supplementary file18 (JPG 96 KB)

Supplementary file19 (JPG 87 KB)

## Data Availability

No datasets were generated or analysed during the current study.
