# Supplementary figures and images for: Robotic versus laparoscopic TME for rectal cancer: meta-analysis of pathological quality indicators
Source: Tech Coloproctol. 2026 May 29;30(1):69. doi: 10.1007/s10151-026-03369-7 (PMC13222314; doi:10.1007/s10151-026-03369-7)

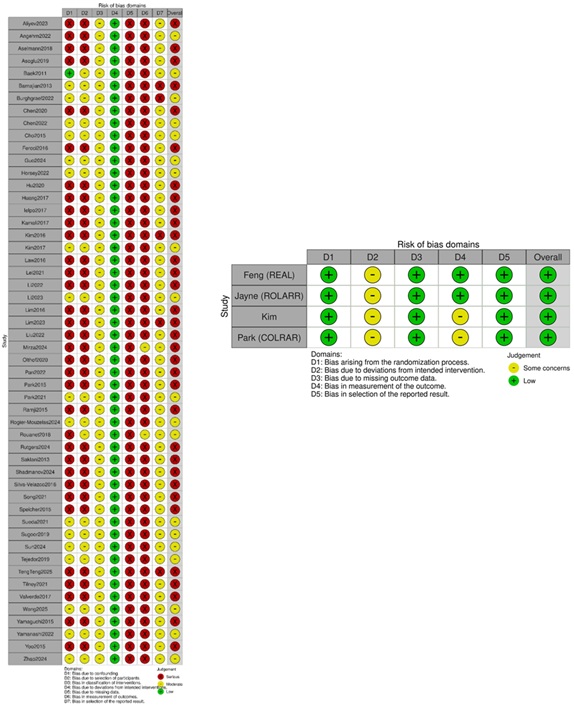

Supplement: Supplementary file 1 — Supplementary file1 (JPG 125 KB) [file 10151_2026_3369_MOESM1_ESM.jpg]

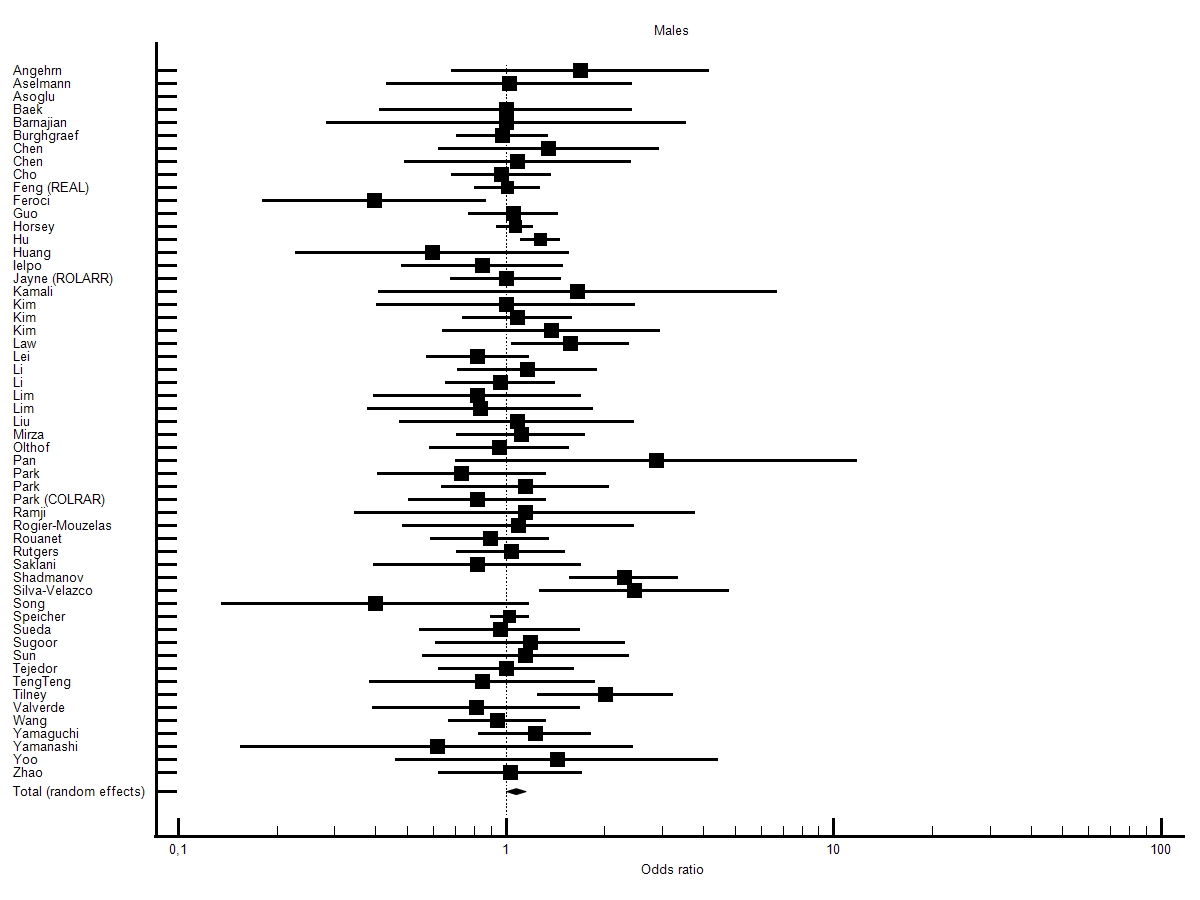

Supplement: Supplementary file 2 — Supplementary file2 (JPG 191 KB) [file 10151_2026_3369_MOESM2_ESM.jpg]

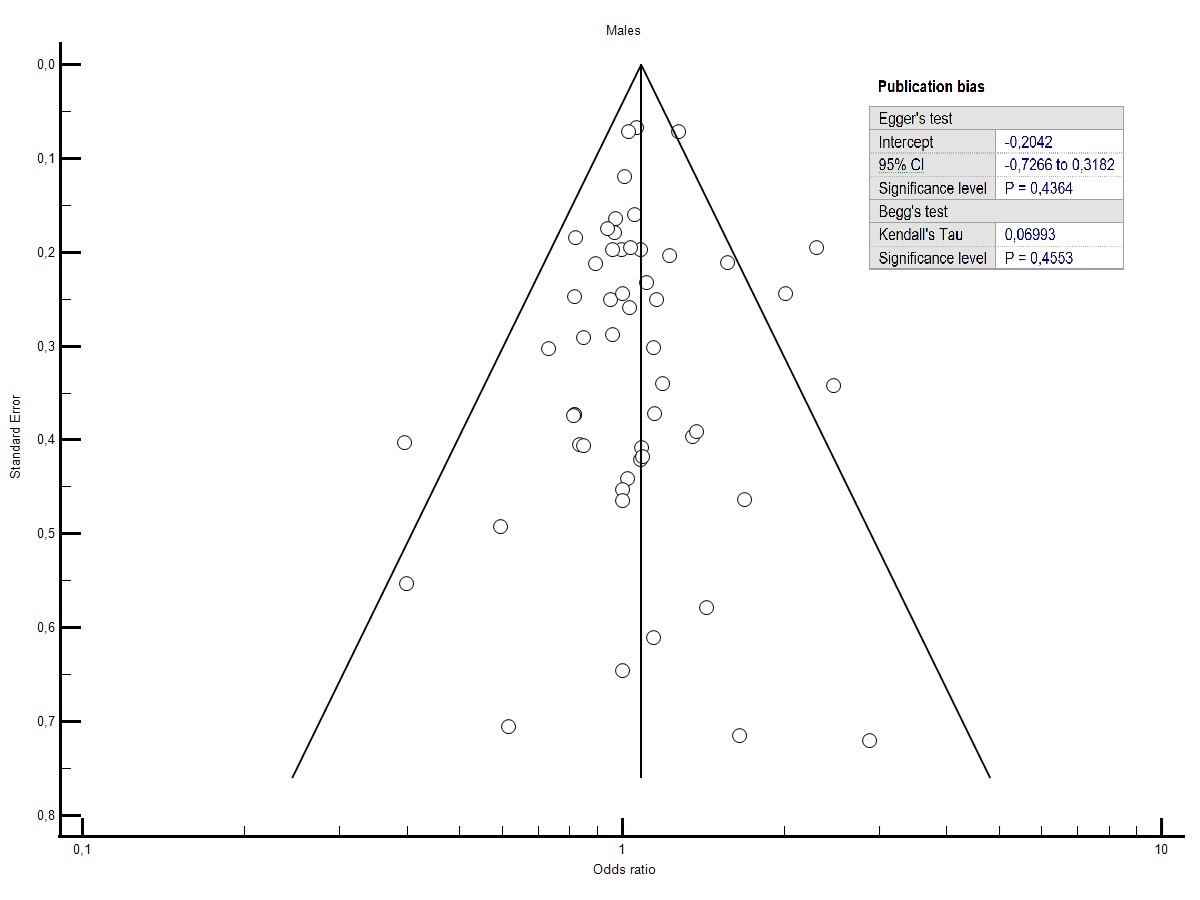

Supplement: Supplementary file 3 — Supplementary file3 (JPG 89 KB) [file 10151_2026_3369_MOESM3_ESM.jpg]

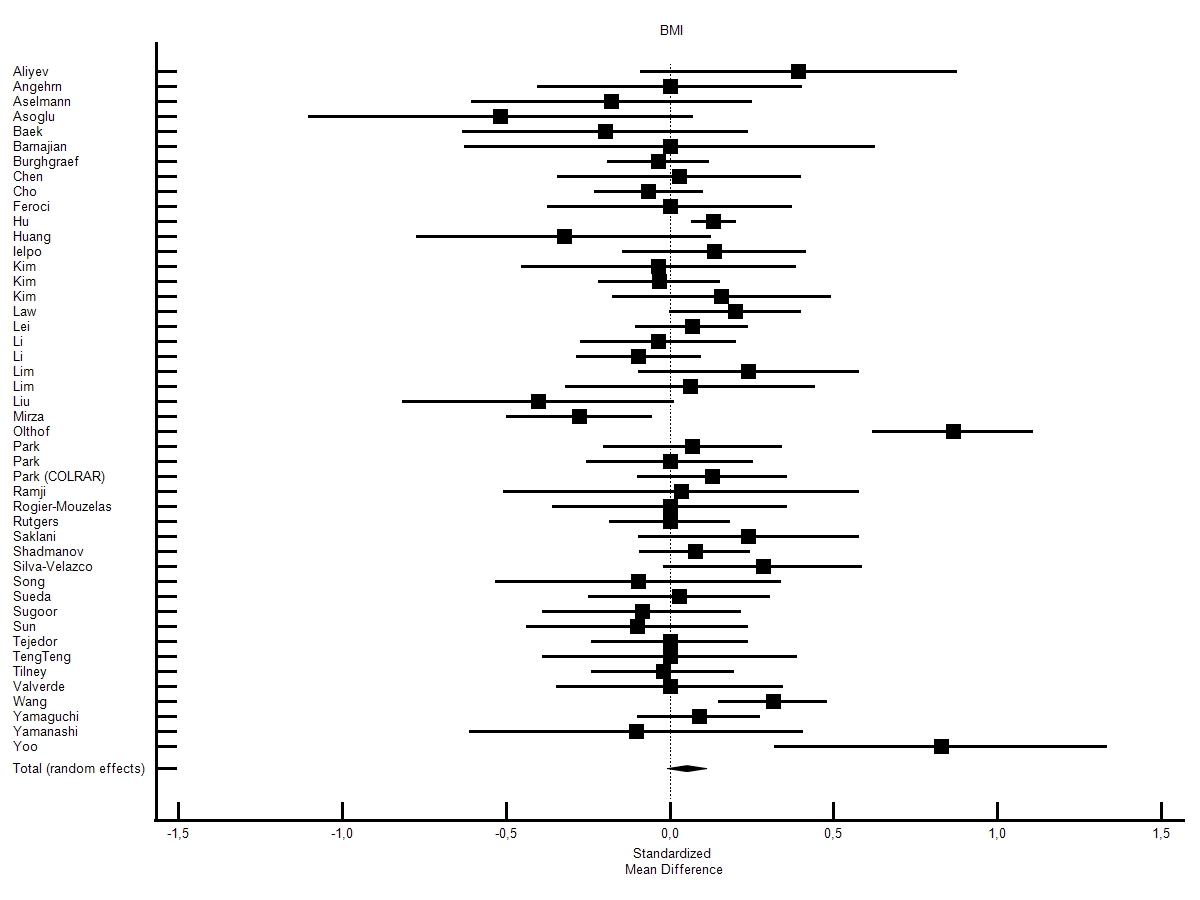

Supplement: Supplementary file 4 — Supplementary file4 (JPG 182 KB) [file 10151_2026_3369_MOESM4_ESM.jpg]

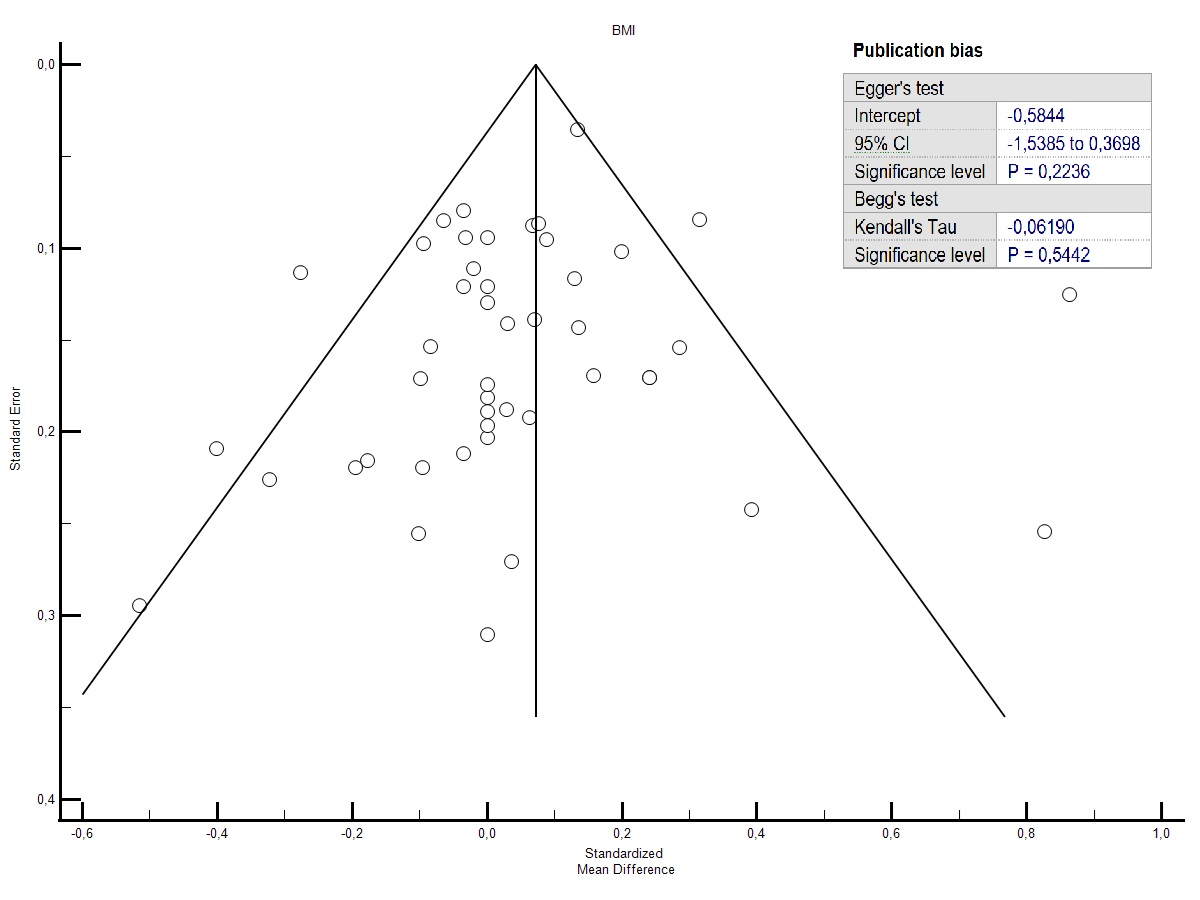

Supplement: Supplementary file 5 — Supplementary file5 (JPG 103 KB) [file 10151_2026_3369_MOESM5_ESM.jpg]

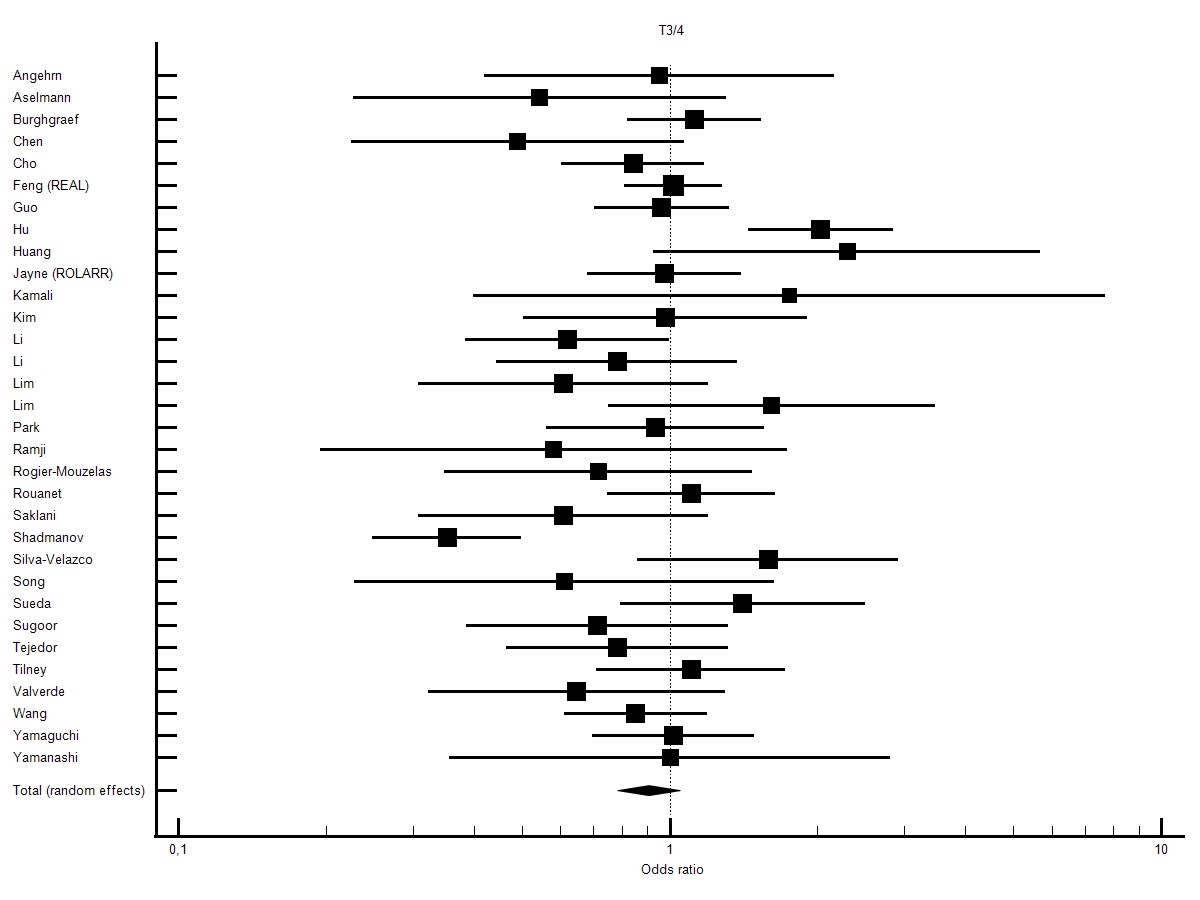

Supplement: Supplementary file 6 — Supplementary file6 (JPG 154 KB) [file 10151_2026_3369_MOESM6_ESM.jpg]

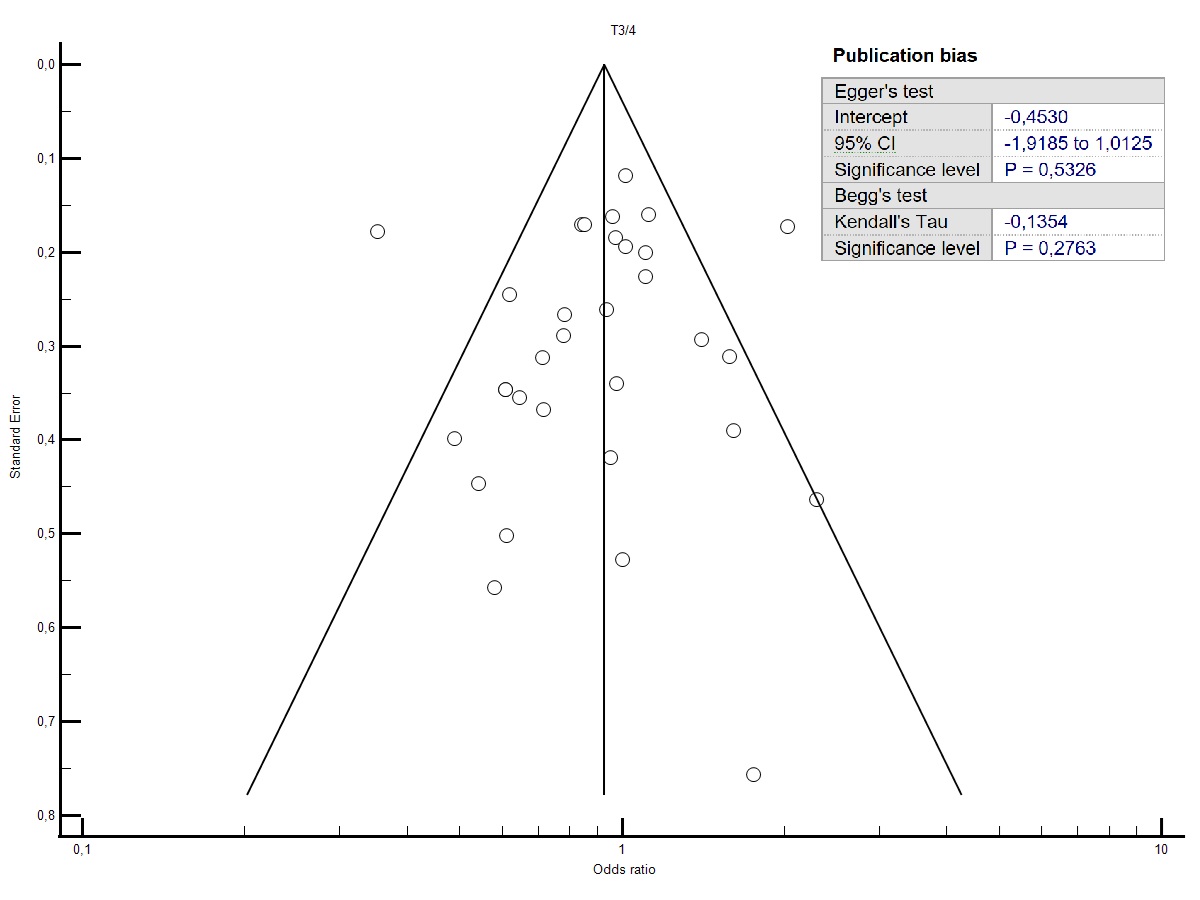

Supplement: Supplementary file 7 — Supplementary file7 (JPG 94 KB) [file 10151_2026_3369_MOESM7_ESM.jpg]

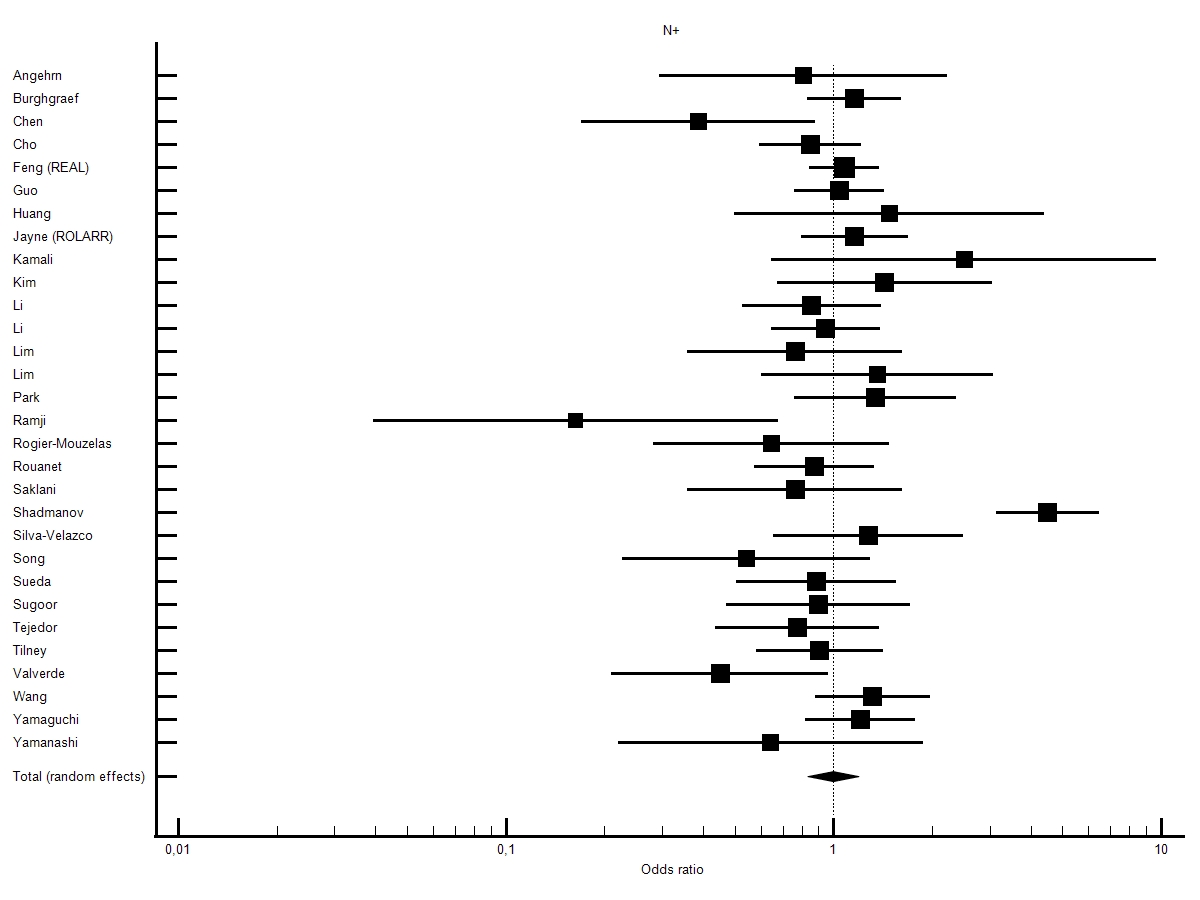

Supplement: Supplementary file 8 — Supplementary file8 (JPG 143 KB) [file 10151_2026_3369_MOESM8_ESM.jpg]

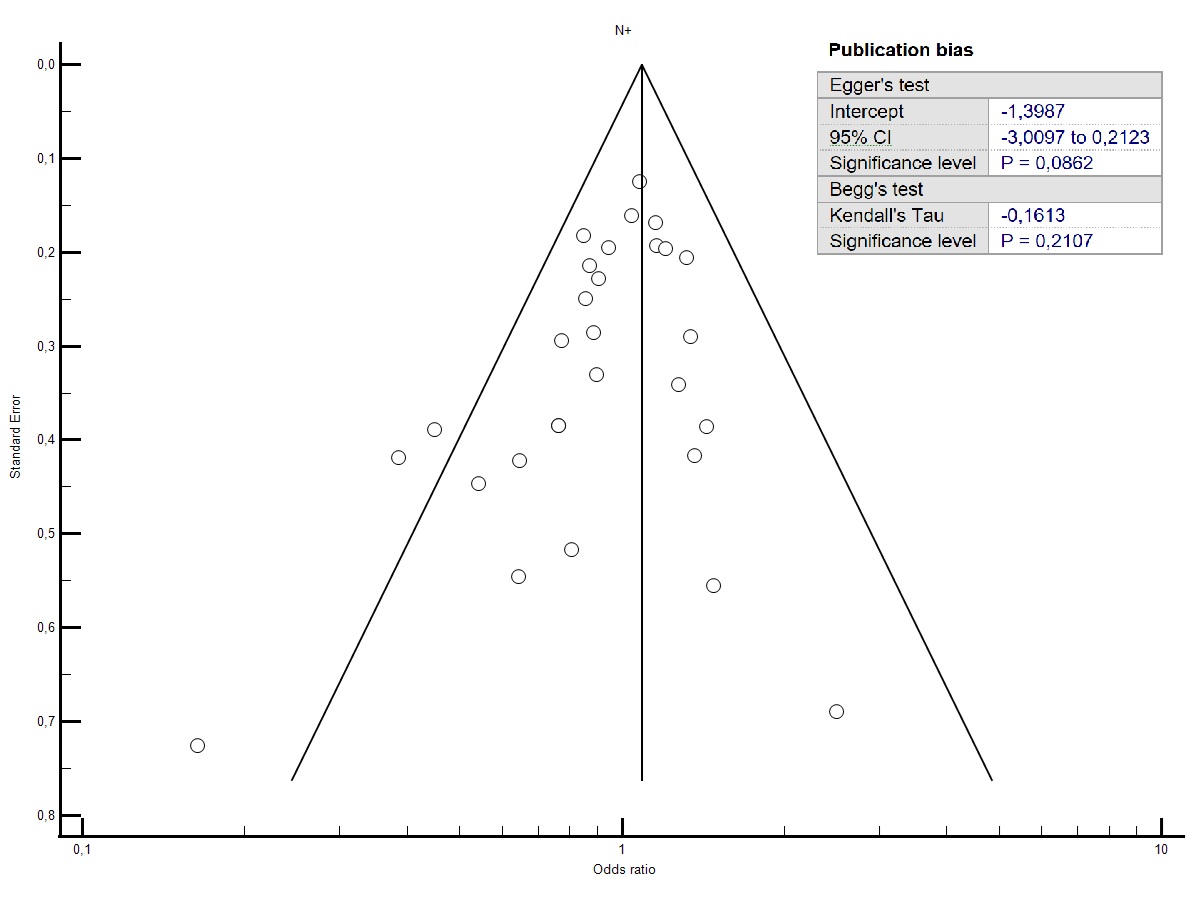

Supplement: Supplementary file 9 — Supplementary file9 (JPG 94 KB) [file 10151_2026_3369_MOESM9_ESM.jpg]

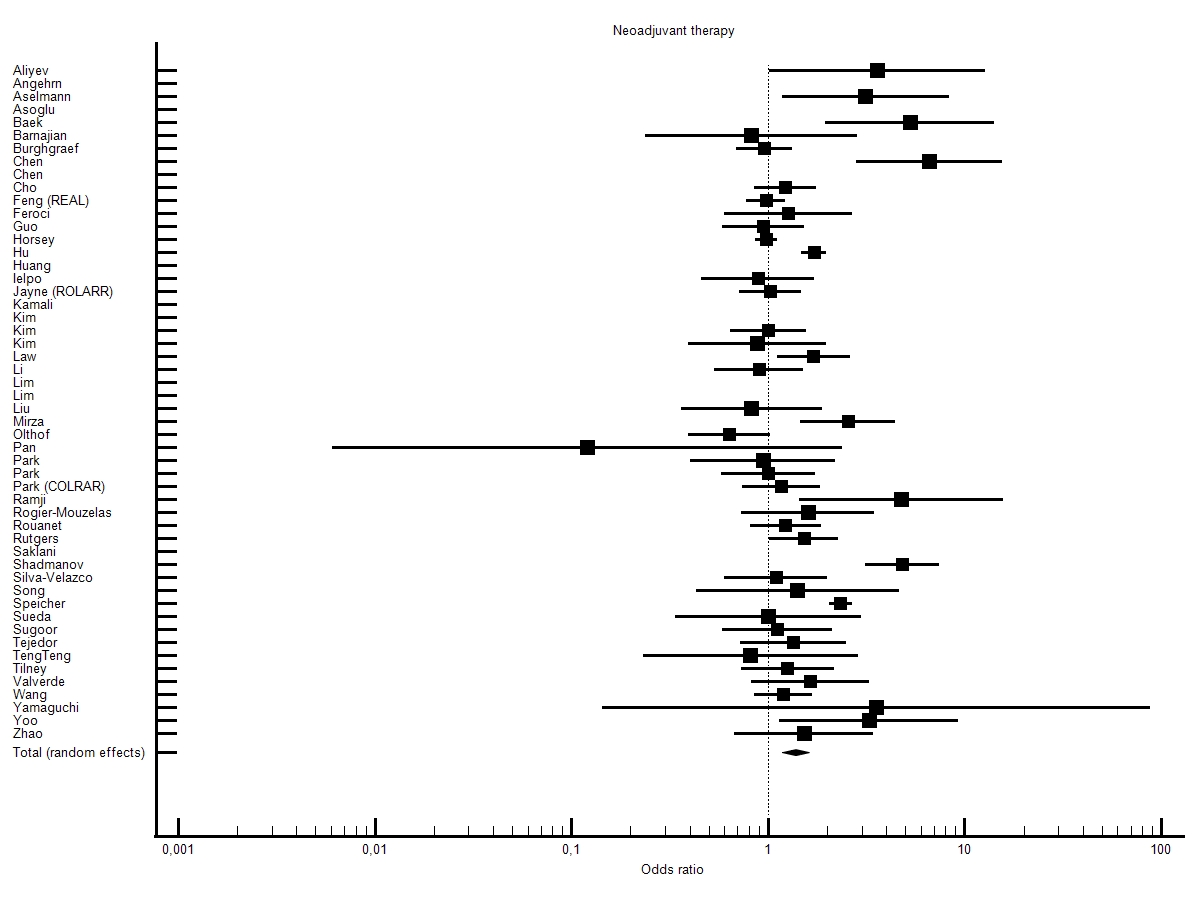

Supplement: Supplementary file 10 — Supplementary file10 (JPG 179 KB) [file 10151_2026_3369_MOESM10_ESM.jpg]

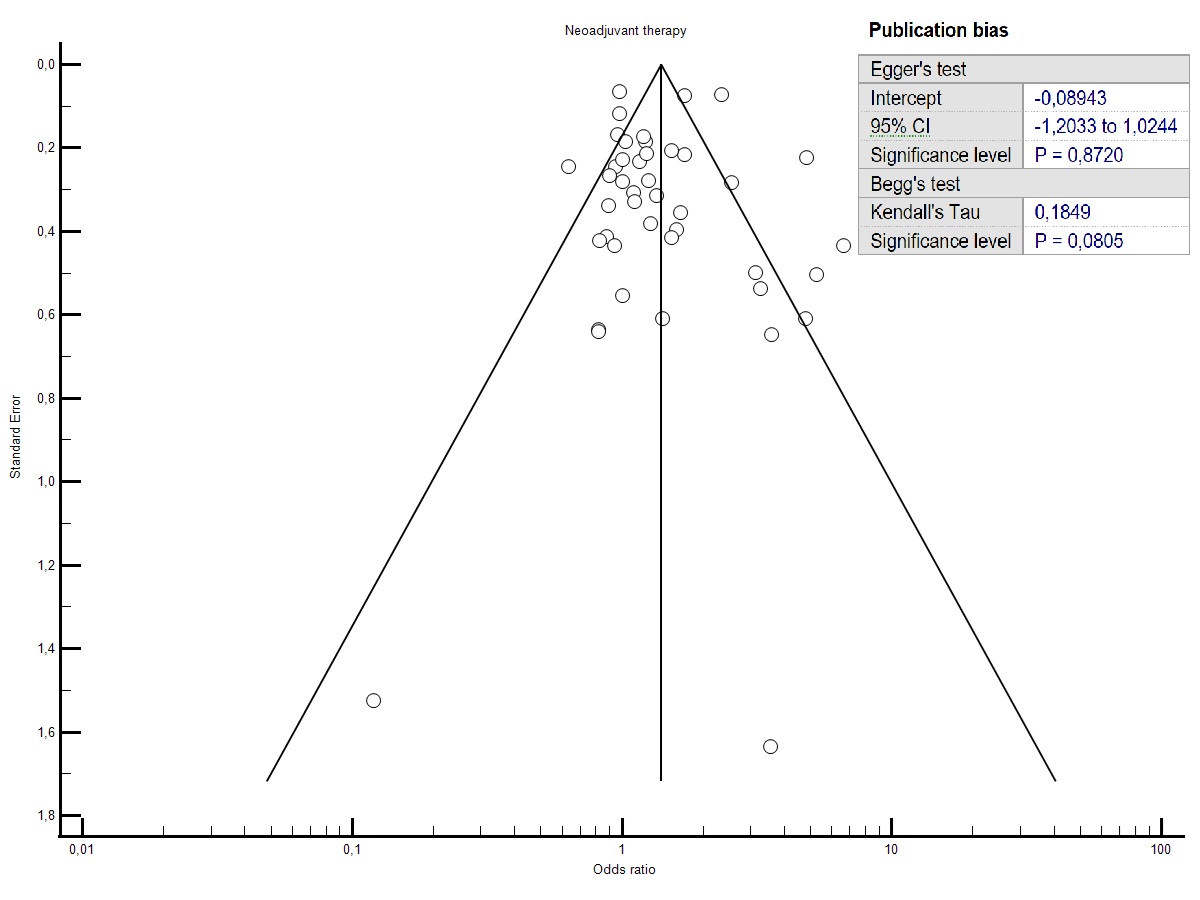

Supplement: Supplementary file 11 — Supplementary file11 (JPG 104 KB) [file 10151_2026_3369_MOESM11_ESM.jpg]

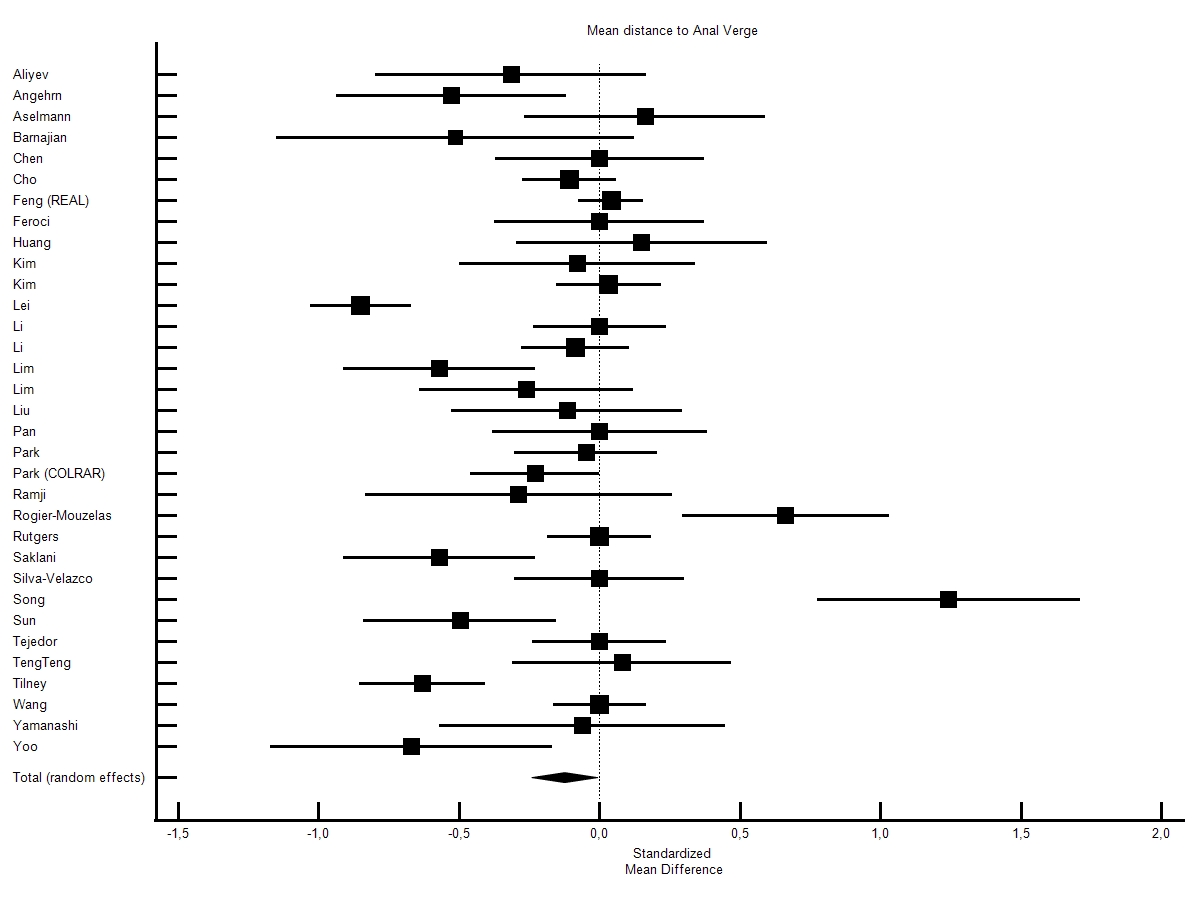

Supplement: Supplementary file 12 — Supplementary file12 (JPG 154 KB) [file 10151_2026_3369_MOESM12_ESM.jpg]

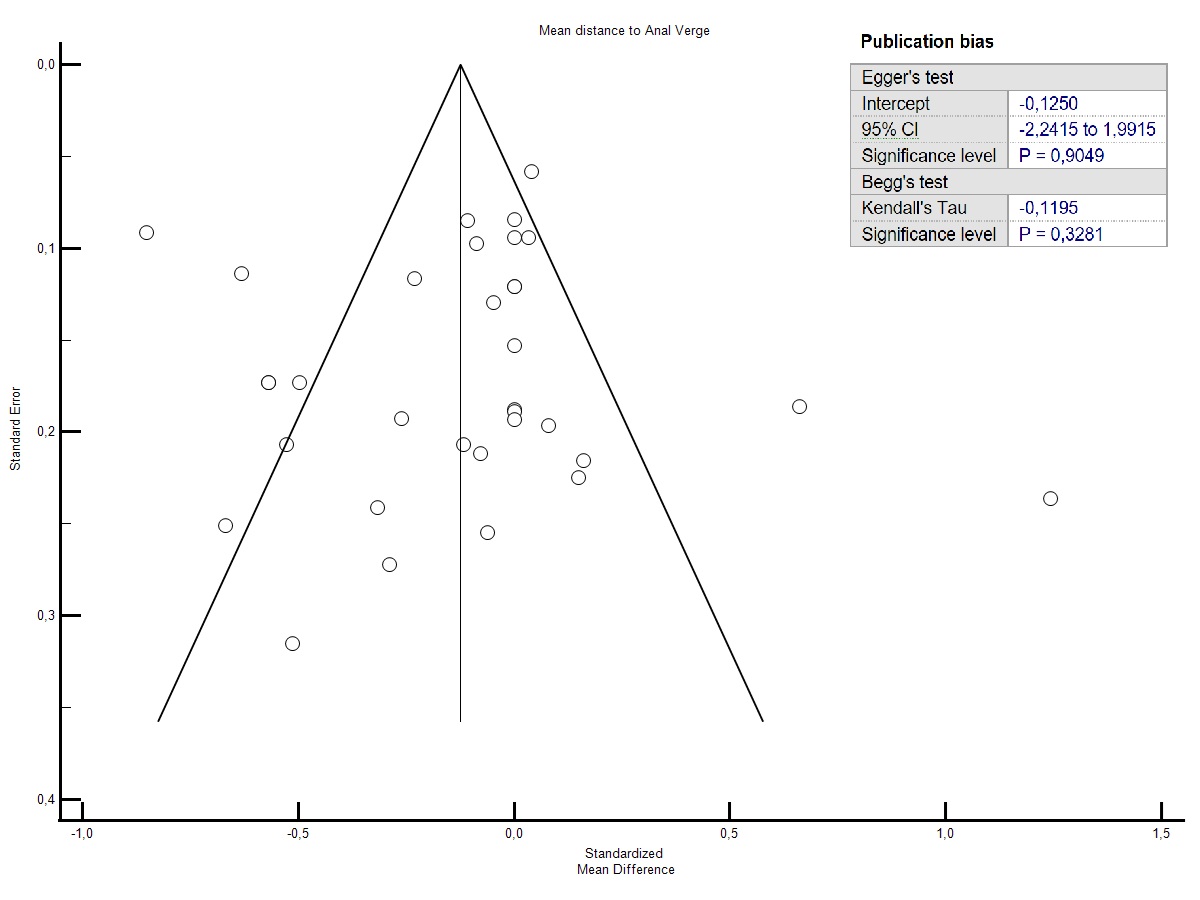

Supplement: Supplementary file 13 — Supplementary file13 (JPG 94 KB) [file 10151_2026_3369_MOESM13_ESM.jpg]

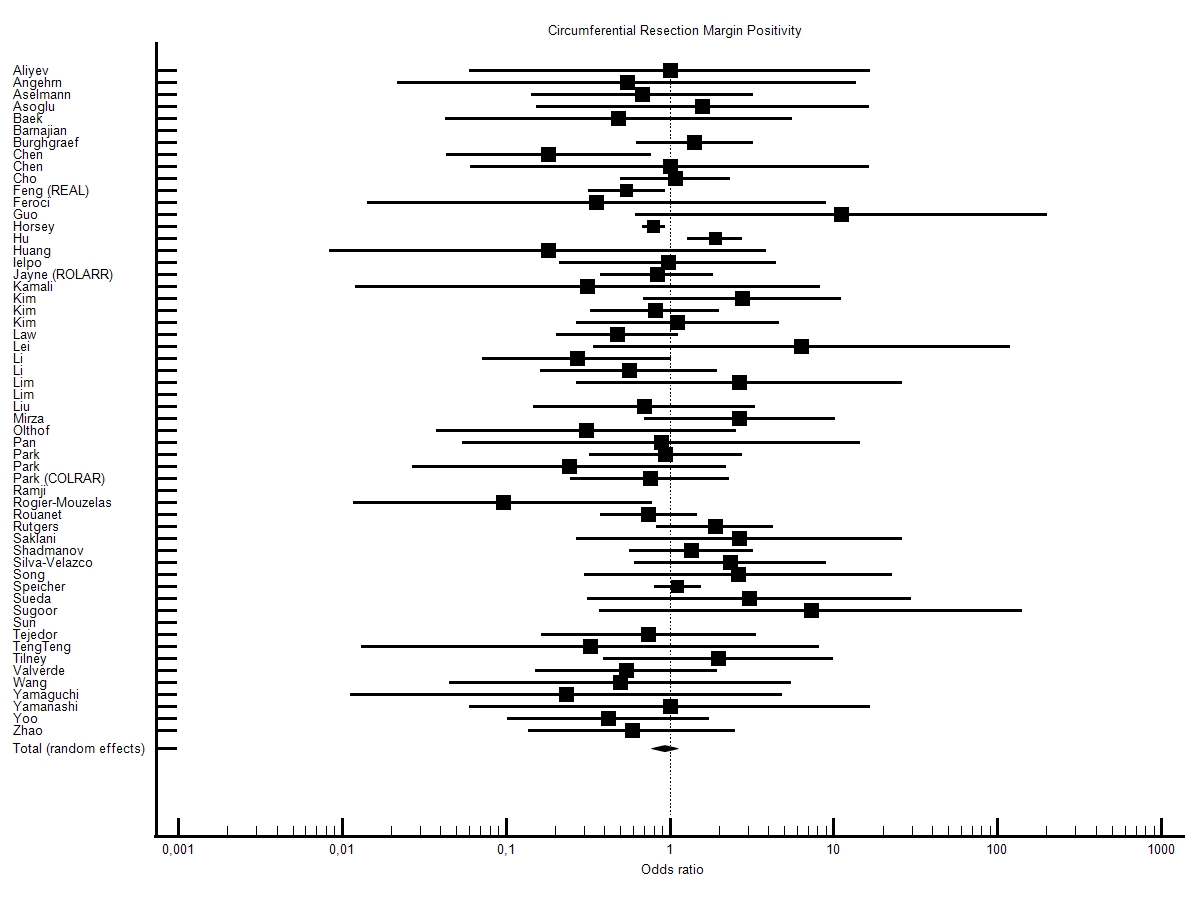

Supplement: Supplementary file 14 — Supplementary file14 (JPG 210 KB) [file 10151_2026_3369_MOESM14_ESM.jpg]

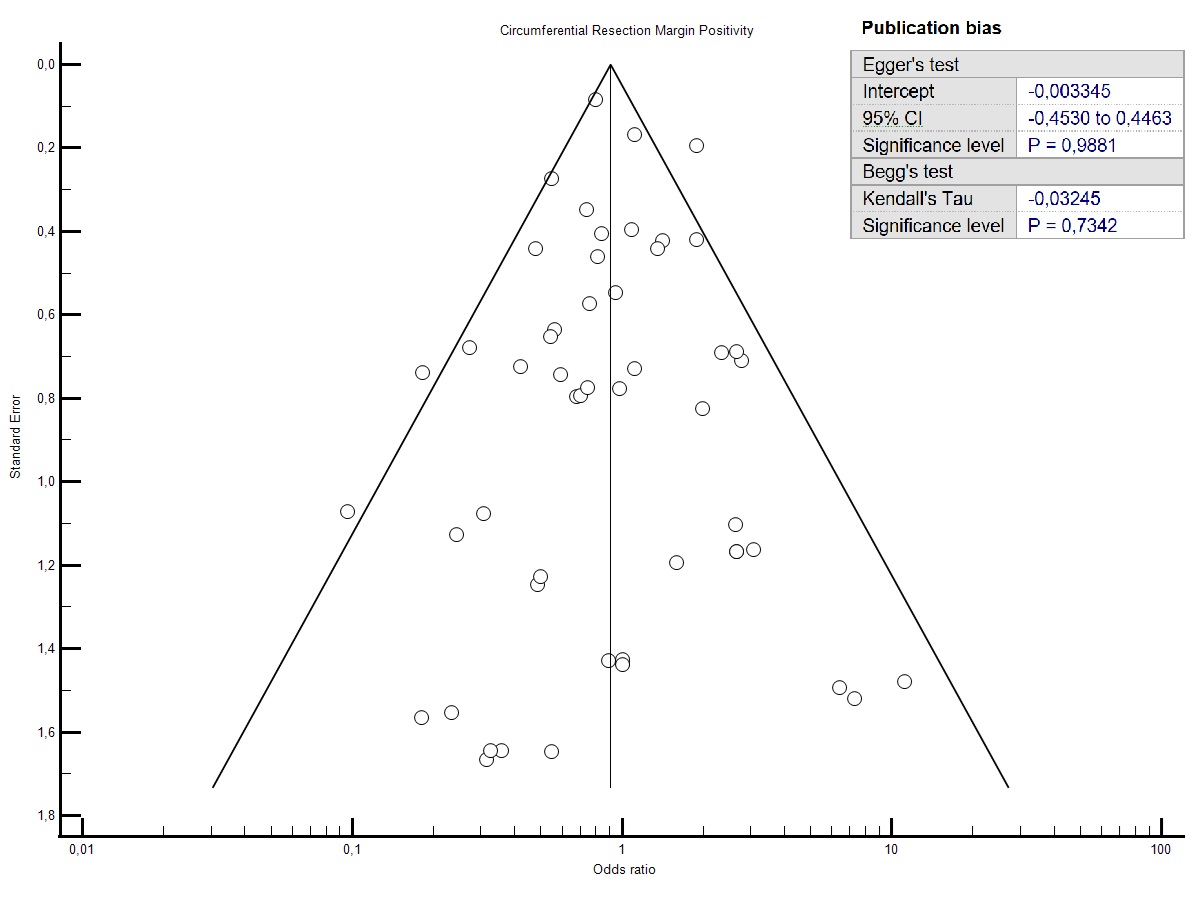

Supplement: Supplementary file 15 — Supplementary file15 (JPG 107 KB) [file 10151_2026_3369_MOESM15_ESM.jpg]

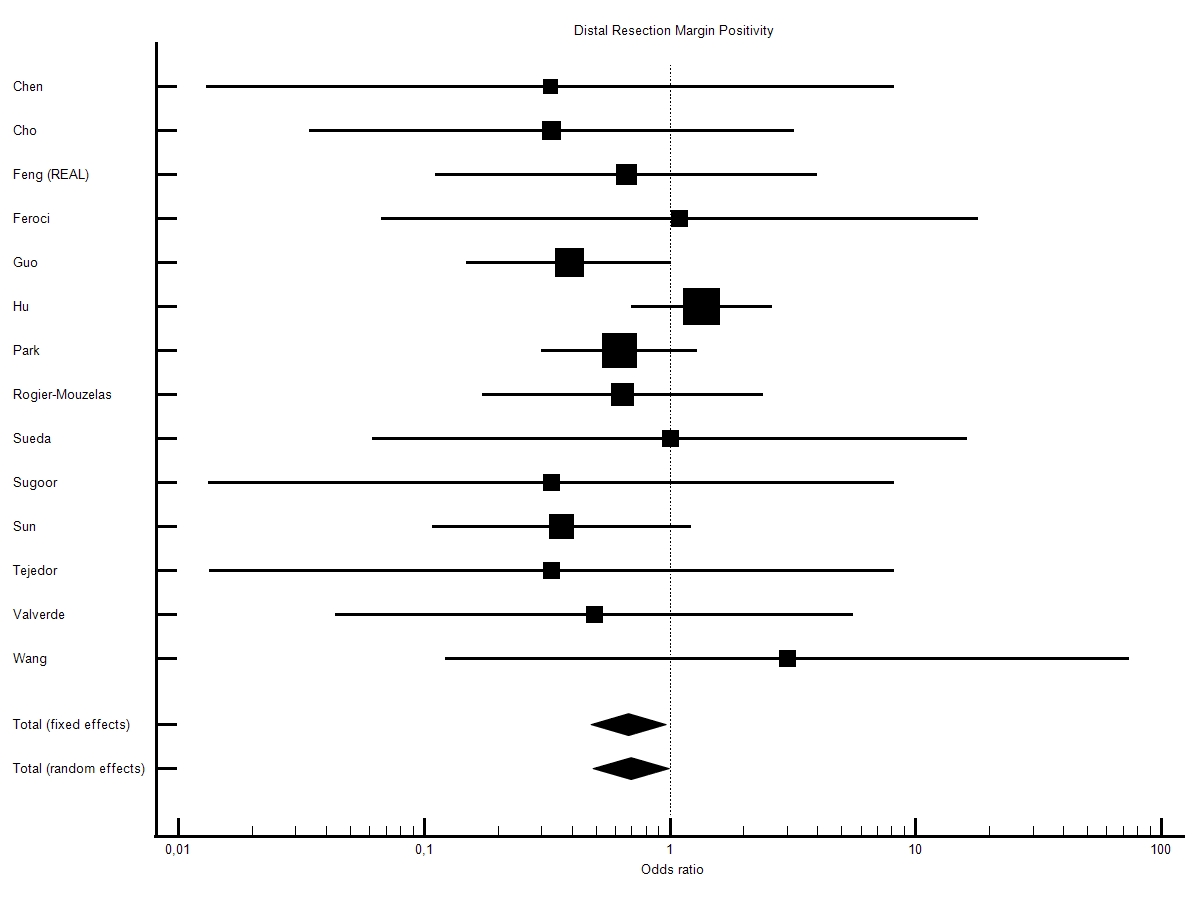

Supplement: Supplementary file 16 — Supplementary file16 (JPG 114 KB) [file 10151_2026_3369_MOESM16_ESM.jpg]

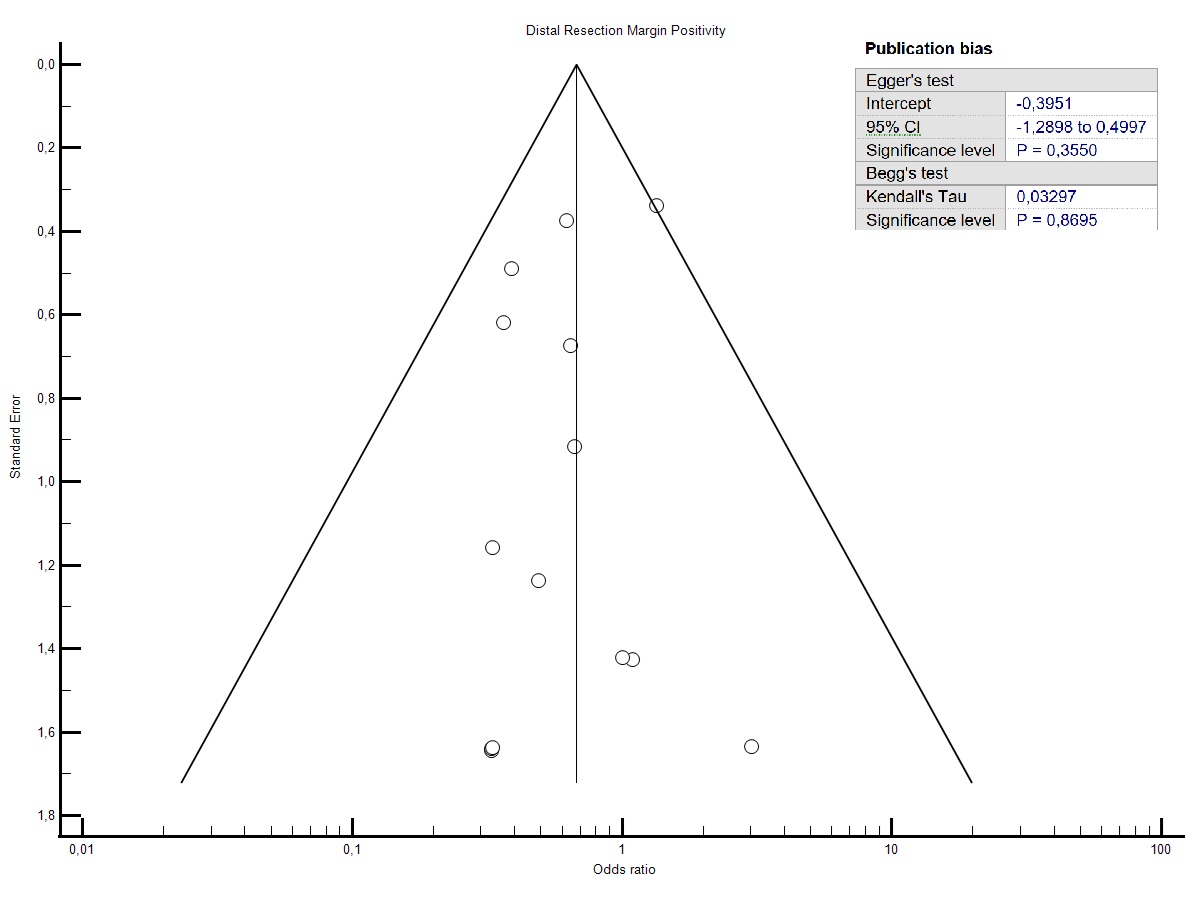

Supplement: Supplementary file 17 — Supplementary file17 (JPG 90 KB) [file 10151_2026_3369_MOESM17_ESM.jpg]

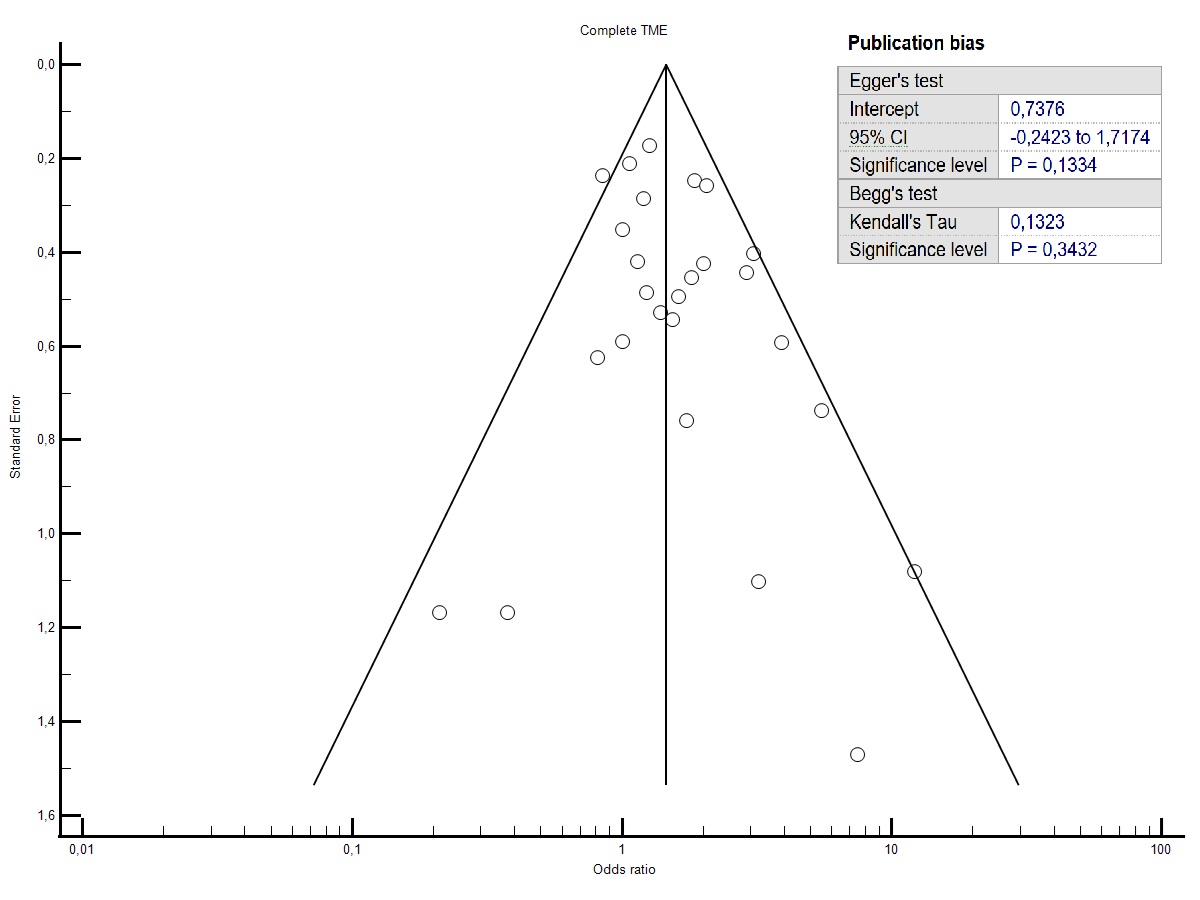

Supplement: Supplementary file 18 — Supplementary file18 (JPG 96 KB) [file 10151_2026_3369_MOESM18_ESM.jpg]

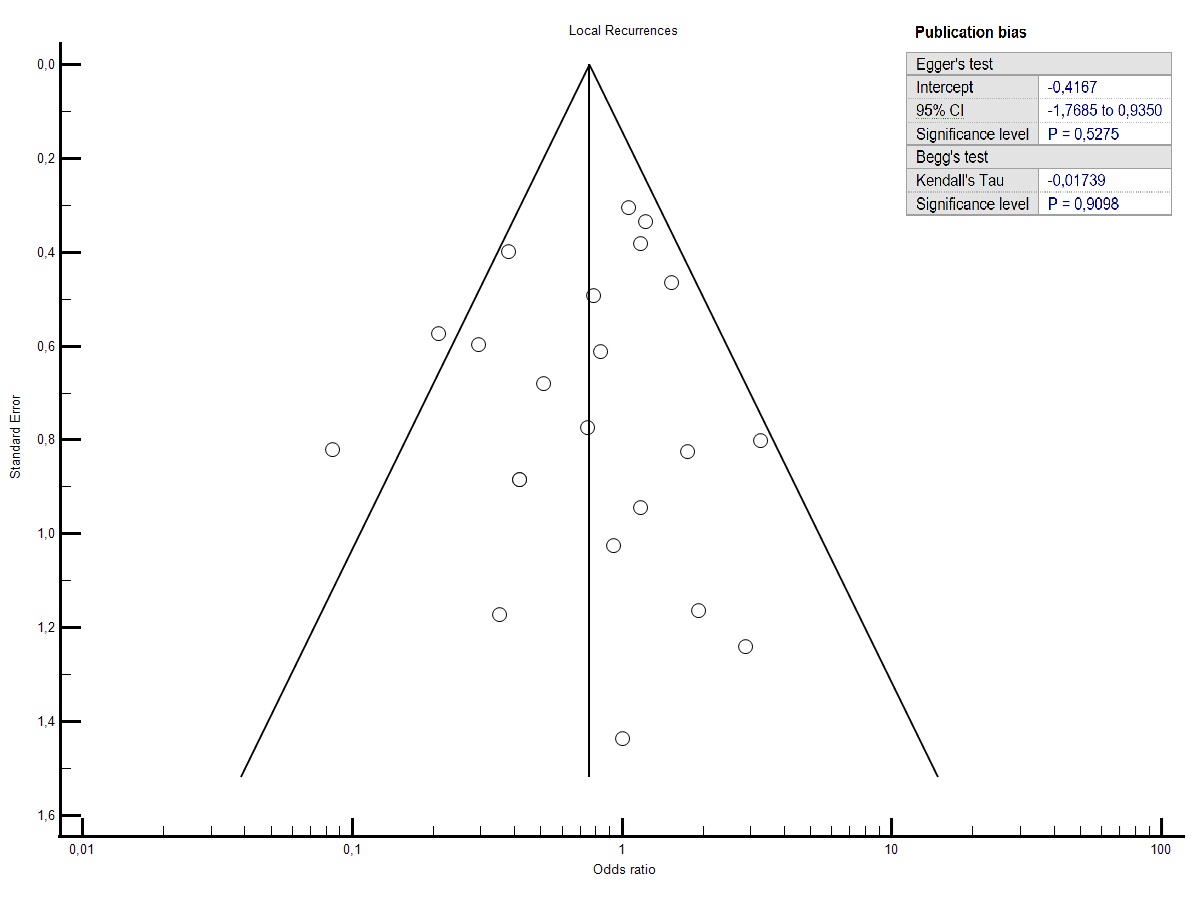

Supplement: Supplementary file 19 — Supplementary file19 (JPG 87 KB) [file 10151_2026_3369_MOESM19_ESM.jpg]
